# Validation of a multiplexed and targeted lipidomics assay for accurate quantification of lipidomes

**DOI:** 10.1016/j.jlr.2022.100218

**Published:** 2022-04-27

**Authors:** Nanyan Rena Zhang, Nathan G. Hatcher, Kim Ekroos, Komal Kedia, Monika Kandebo, Jacob N. Marcus, Sean M. Smith, Kevin P. Bateman, Daniel S. Spellman

**Affiliations:** 1 Department of Discovery, Preclinical and Translational Medicine, Merck & Co., Inc., West Point, PA, USA; 2 Department of Neuroscience, Merck & Co., Inc., West Point, PA, USA; 3 Lipidomics Consulting Ltd, Esbo, Finland

**Keywords:** quantitative lipidomics, targeted lipidomics, hydrophilic interaction chromatography (HILIC), normal phase liquid chromatography (NPLC), triple quadrupole mass spectrometry, scheduled MRM, glucosylceramide synthase, FDA bioanalytical method validation guidance for industry, Benzoxazole 1, glycosphingolipid metabolism, BA, bioanalytical, BSA, bovine serum albumin, Cer, ceramide, DAG, diacylglycerol, DB, double bond, DCM, dichloromethane, FA, fatty acyl, FDA, food and drug administration, GalCer, galactosyl-ceramide, GCS, glucosylceramide synthase, GlcCer, glucosyl-ceramide, HexCer, hexosylceramide, HILIC, hydrophilic interaction chromatography, IPA, 2-propanol, IS, internal standard, ISF, in-source fragmentation, LOQ, limit of quantitation, NPLC, normal phase chromatography, PC, phosphatidylcholine, PE, phosphatidylethanolamine, PG, phosphatidylglycerol, QqQ, triple quadrupole, RT, retention time, SIL, stable isotope labeled, SM, sphingomyelin, UHPSFC, ultrahigh-performance supercritical fluid chromatography, ULOQ, upper limits of quantitation

## Abstract

A major challenge of lipidomics is to determine and quantify the precise content of complex lipidomes to the exact lipid molecular species. Often, multiple methods are needed to achieve sufficient lipidomic coverage to make these determinations. Multiplexed targeted assays offer a practical alternative to enable quantitative lipidomics amenable to quality control standards within a scalable platform. Herein, we developed a multiplexed normal phase liquid chromatography-hydrophilic interaction chromatography multiple reaction monitoring method that quantifies lipid molecular species across over 20 lipid classes spanning wide polarities in a single 20-min run. Analytical challenges such as in-source fragmentation, isomer separations, and concentration dynamics were addressed to ensure confidence in selectivity, quantification, and reproducibility. Utilizing multiple MS/MS product ions per lipid species not only improved the confidence of lipid identification but also enabled the determination of relative abundances of positional isomers in samples. Lipid class-based calibration curves were applied to interpolate lipid concentrations and guide sample dilution. Analytical validation was performed following FDA Bioanalytical Method Validation Guidance for Industry. We report repeatable and robust quantitation of 900 lipid species measured in NIST-SRM-1950 plasma, with over 700 lipids achieving inter-assay variability below 25%. To demonstrate proof of concept for biomarker discovery, we analyzed plasma from mice treated with a glucosylceramide synthase inhibitor, benzoxazole 1. We observed expected reductions in glucosylceramide levels in treated animals but, more notably, identified novel lipid biomarker candidates from the plasma lipidome. These data highlight the utility of this qualified lipidomic platform for enabling biological discovery.

## Graphical abstract

**Figure:**
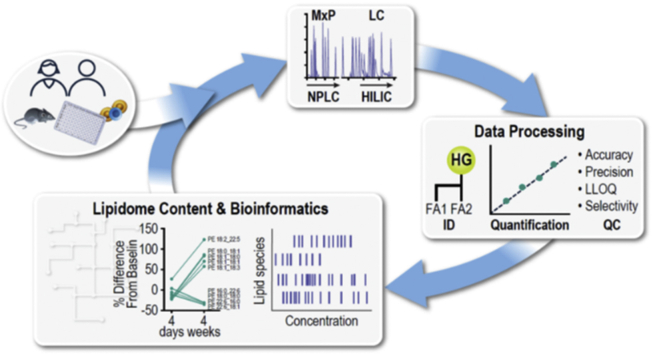

Lipids are vital biomolecules serving fundamental roles in cellular signaling, cell membrane architecture, energy storage, and metabolism. Seemingly minor structural differences among individual lipid species, such as the number, position, and geometry of double bonds (DBs) in acyl chains, are pivotal determinants of their functions (1). Alterations in lipid metabolic network may trigger a cascade of deleterious cellular events. The increasing evidence of the biological relevance of lipids and their roles in diseases such as neurodegenerative (2), cancer (3), cardiovascular (4), and infectious diseases (5) during the past decade, has expanded the development of lipidomics for use in biomedical research (6). Analytical methods that accurately quantify lipid molecular species in biological samples are pivotal to enable understanding of underlying biological mechanisms of disease pathology at a deeper level and offer a potential to accelerate the development of new therapeutics.

Lipids have been classified into eight major categories in the LIPID MAPS repository (7) based on their building blocks. From analytical and physiochemical standpoints, neutral lipids are hydrophobic molecules lacking charged groups. In contrast, polar lipids have variety of polar headgroups. Both types share the complexities in fatty acyl (FA) chains including but not limited to chain lengths and positions of DBs. In addition, a large variation in stereochemistry also exists, such as epimers (e.g., glucosyl- and galactosyl-ceramide (GlcCer and GalCer)) and regioisomers (e.g., *sn*-1 and *sn*-2 acyl position of phospholipids). This structural heterogeneity generates a large number of distinct species, which contributes to an extremely complex lipidomic chemical space (8). Eukaryotic cells may contain thousands of distinct species with their lipid concentrations varying between organelles up to several million fold (9). All these factors pose extreme challenges to the analytics required to achieve precise elucidation and quantification of distinct lipid species.

Despite significant technological and methodological advances over the past decade, determining the precise content of a complex lipidome at the level of exact molecular species remains challenging. For example, lipid extraction methods that not only generate clean, enriched samples benefiting MS detection but also protect lipids from degradation and oxidation are needed (10). Similarly, there is a need for more confident data processing solutions especially for untargeted lipidomics data. The availability of lipid standards, especially stable isotope labeled (SIL) standards, determines the buildup of quantitative assays. In fact, standardization in lipidomics methods (11) and lipid nomenclature (12) has been initiated to address the discrepancies in quantitative information of existing methodologies and lipid annotation (13). Typically, quantification is performed using one nonendogenous internal standard (IS) per lipid subclass (14). A requirement and standard practice of industrial bioanalytical methods is to interpolate unknown concentrations against valid calibration curves. This is a necessity along with preset acceptance criteria in quality control samples according to the Food and Drug Administration (FDA) Bioanalytical (BA) Validation Guidance (15) to ensure robust quantitation. In an idealized assay, a practical analytical workflow would align with ongoing lipidomics standardization (11) to assure high quality data reported in a consistent manner.

To date, quantitative lipidomics is achieved by either direct infusion or chromatography-based MS approaches. It is widely recognized that coionization of endogenous analytes and their ISs must be accounted for using MS-based quantification (16). Shotgun lipidomics satisfies this norm of quantification, and with its steady performance and high throughput, it becomes attractive in many ways (17, 18). However, the simultaneous injection of lipid ions by direct infusion complicates the ionization process resulting in compromised detection of ions with either low abundances or weak ionization efficiencies. MS spectra interpretation is challenging and limited because of a high degree of overlapping signals. Chromatography separation can effectively reduce this complexity. Reversed-phase liquid chromatography provides superior separation (19) based on the apolar properties residing in FA chains. However, it is not preferred for quantification, mostly due to the lack of SIL lipid ISs with various FA chains. Hydrophilic interaction chromatography (HILIC) and normal phase chromatography (NPLC) separate lipids primarily based on lipid classes. This enables a quantification strategy using a few representative lipid standards and SIL ISs. Still, HILIC and NPLC have separation limitations as HILIC is relatively less capable of resolving nonpolar lipids while NPLC is suboptimal in resolving polar lipids within a relatively short eluting period (e.g., in less than 15–20 min).

Here, we seek an easily implementable workflow in line with FDA BA guidance and ongoing lipidomics standardization to generate quantitative lipidomics data to enable acceptable turnaround of robust lipidomic data sets. The method couples multiplexed NPLC-HILIC separation and fast scanning triple quadrupole (QqQ) MS employing Multiple Reaction Monitoring (MRM) detection within a 20-min run time per sample. The method is capable of providing deep structural and accurate quantitative information of lipidome content with resolution down to positional isomers of molecular species (8). Applying this method, we report lipidome-wide alterations in a highly specific and well-studied pharmacological model by analyzing plasma of mice treated with a potent glucosylceramide synthase (GCS) inhibitor Benzoxazole 1 (BZ1). These data suggest that alterations of single points within lipid metabolic pathways can have broad effects across the lipidome that remain to be explored.

## Materials and methods

### Chemicals, reagents, and materials

HPLC-grade water, acetonitrile, methanol, chloroform, hexane, acetone, and dichloromethane (DCM) were obtained from Thermo Fisher Scientific (Fair Lawn, NJ). Formic acid, acetic acid, ammonium acetate, 2,6-Di-tert-butyl-4-methyphenol, 2-propanol (IPA), and bovine serum albumin (BSA) were purchased from Sigma-Aldrich (St. Louis, MO). SRM 1950 human plasma was obtained from National Institute of Standards and Technology (Gaithersburg, Maryland). Most lipid standards (supplemental Table S1) were products of Avanti Polar Lipids (Alabaster, AL) at the time of the experiment. 1x PBS buffer was prepared using PBS tablets from MiliporeSigma (Burlington, MA). BZ1 was synthesized by MSD chemists. Wheaton 4-ml and 20-ml amber glass vials with PTFF liner screw caps were used for lipid working solution preparations. Lipid extraction plate (2.0 ml glass conical insert in 96-well vial loader) was obtained from Chrom Tech (Apple Valley, MN). LC/MS injection plate (amber 1 ml low carry tapered glass inserts in 96-well plate with pre-slit cap mat) was from Analytical Sales & Services, Inc (Flanders, NJ). Various Rainin single and multi-channel pipettes and matching pipette tips were used for all manual liquid aliquoting. A Microlab Nimbus workstation equipped with a CORE 96 Probe Head (Hamilton, Reno, Nevada) was used for lipid extraction. A 96-well solvent evaporator device SPE Dry-96 was obtained from Jones Chromatography (Lakewood, CO) and connected to an in-house Nitrogen source. A Multi-Tube vortexer from VRM Scientific (Radnor, PA) was used for sample mixing.

### Inhibition of GCS in mice

Heterozygous GBA1 D409V mice (C57BL/6N-Gba tm1.1 Mjff/J, stock number: 019106, Jackson Laboratory, Bar Harbor, ME) were rederived, bred, and maintained at Taconic. Animals were acclimated to housing for at least one week from delivery and kept on a normal 12 h/12 h light/dark cycle for a week or longer during which time access to food and water were provided ad libitum. Mice were divided into separate groups and fed chow formulated with a potent GCS inhibitor BZ1 or matching control chow for four days or four weeks. BZ1 content in chow (Rodent diet with 10 kcal% Fat and 220 mg of BZ1 API/kg; Research Diets, Inc., New Brunswick, NJ) was selected to target chronic exposure levels of unbound BZ1 approximating two times the reported GCS enzyme IC50 value of 18 nM (20). Weight of chow consumed was measured daily across individuals to estimate BZ1 daily doses and to verify similar feeding rates between mice-fed control versus BZ1-formulated diets. At the conclusion of treatment, animals were anesthetized with isoflurane (1–4% in oxygen at 2 l/minute) and blood was collected via cardiac puncture immediately prior to euthanasia via decapitation. Plasma was prepared and stored at −70°C until lipid extraction and analyses. All animal studies were performed under the approval of the Merck & Co., Inc., Kenilworth, NJ, Institutional Animal Care and Use Committee.

### Calibration standards and internal standards preparation

A lipid standard mixture and a respective deuterated IS mixture were prepared. Ten calibration working solutions were prepared by serial diluting the standard mixture in DCM:IPA (1:1, v/v). The calibration standards were prepared by spiking the calibration working solutions into 2% BSA according to supplemental Tables S3 and S4 for method validation. The calibration curves were constructed by nominal concentrations of lipid calibrators (X-axis) and MS peak area ratios of the lipid standard over IS (Y-axis).

### Lipid extraction

To 20 μl mice plasma study samples, calibrators, and 2% BSA (single blanks), 10 μl IS mixture was spiked (supplemental Table S4). Ten microliters DCM:IPA (1:1, v/v) was spiked to several 20 μl 2% BSA solutions as the double blanks. Two hundred microliter ice-cold methanol containing 0.1% 2,6-Di-tert-butyl-4-methyphenol was added to all samples. The plate was mixed by vortexing gently for 2 min. Hamilton Microlab Nimbus was programmed to dispense 450 μl chloroform and 120 μl 20 mM acetic acid into each of the wells. The plate was mixed vigorously for 30 min. Phase separation was performed by centrifugation (Sorvall Legend centrifuge, Kendro Laboratory, Germany) at 2500 rpm for 10 min at 15°C. Three hundred sixty microliters of lower phase was transferred to a 1 ml glass LC/MS injection plate and dried under nitrogen gas at room temperature. To the remaining upper aqueous phase, 360 μl of chloroform was added and repeated as above. After centrifugation, the lower organic phase was pooled with the previous organic fraction. The final sample extracts were dried under nitrogen and reconstituted with 150 μl IPA:DCM (1:1, v/v) prior to analyses.

### LC-MRM analysis

**Fig. 1:**
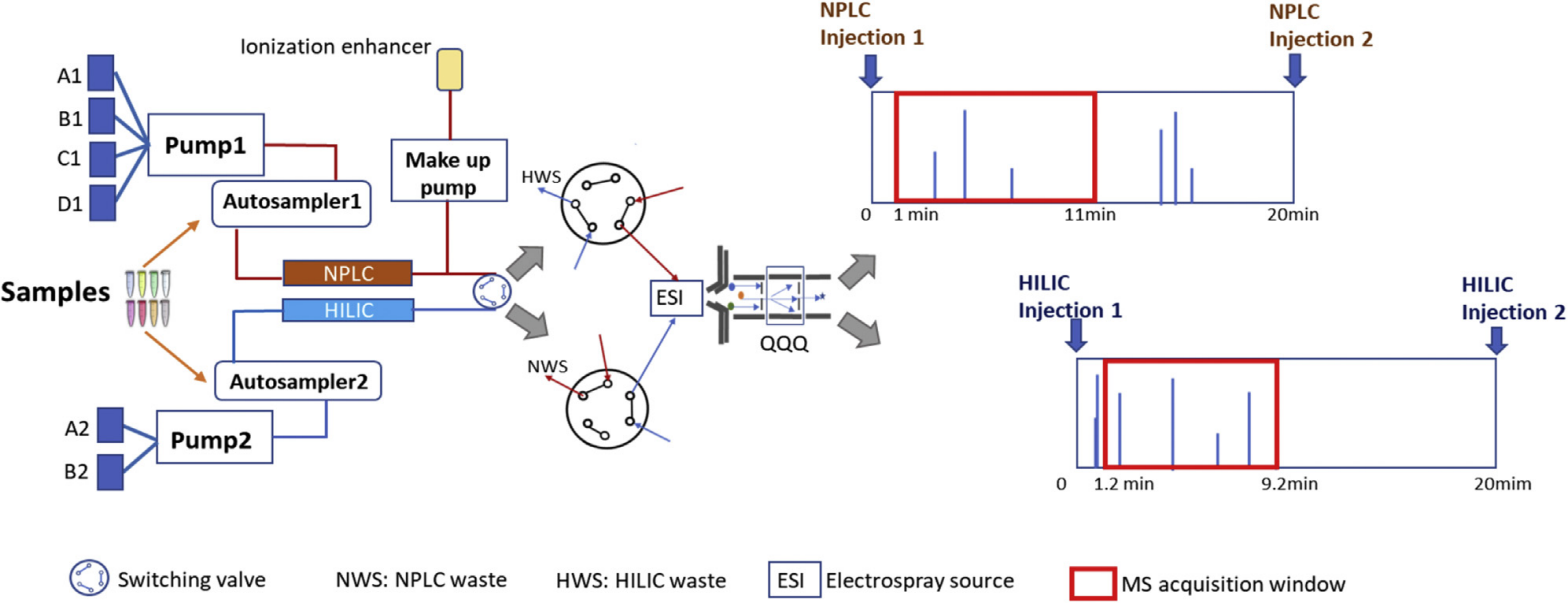
Schematic diagram of the multiplexed NPLC/HILIC-QqQ lipidomics setup. HILIC, hydrophilic interaction chromatography; NPLC, normal phase chromatography; QqQ, triple quadrupole.

The schematic diagram of the multiplexed LC-MRM setup is shown in Fig. 1. A duo channel UPLC system (Thermo Scientific Waltham, MA) equipped with two fast gradient quaternary pumps and two autosamplers was coupled to a QqQ MS (Sciex API 6500, Framingham, MA). Aria software was used to schedule staggered NPLC and HILIC separations. Aria also managed the QqQ MS to acquire only the selected portions of entire HILIC and NPLC separations. A makeup pump was integrated to mix an ionization enhancing solvent (90% IPA containing 10 mM ammonium acetate and 0.1% formic acid) with the NPLC eluent. The separation columns, mobile phases, and representative gradient conditions are shown in supplemental Table S5.

The QqQ MS was operated using electrospray ionization with the following parameters: curtain gas 40, ion source gas (1, 2) 80, source temperature 295°C, and collision gas (CAD) 9. Other parameters for positive and negative modes are as follows, respectively: ion spray voltage 5500 V and -4500 V, entrance potential 10V and -10V, and collision cell exit potential 15V and -15V.

A library containing thousands of MRM transitions was constructed based on lipid-class specifics as described in supplemental Table S2. Scheduled MRM conditions were optimized based on the retention times (RTs) of synthetic standards. The MRM detection window was set between 40 to 120 s to bracket lipid peaks of the same class. Roughly 2.8 scan cycles per second was used by adjusting target scan time between 0.15 to 0.18 s. The peak integration was performed using MultiQuant (Version 3.03, Sciex). Isotopic correction of the measured lipids was performed using LICAR (21).

### Method validation

Extraction recovery was based on measuring the difference between pre-extraction and post-extraction spiked deuterated lipid standards normalized to selected endogenous lipids. Extraction recovery was assessed using 46 deuterated synthetic lipid standards (see details in supplemental Table S6).

Three calibration curves were tested in parallel to determine the linearity, dynamic range, limit of quantitation (LOQ), and limit of detection. We defined an acceptable calibration curve with a regression coefficient *R*^2^ > 0.99 and minimum six calibrators. We typically define tiered stringencies in precision and accuracy for exploratory measurement versus assays for use within FDA-regulated requirements. Early discovery biomarker analyses require less stringency in terms of acceptance criteria in order to reduce the number of false negative responses in initial analyses prior to follow-up validation. In this case, each calibrator, including limit of quantification, must meet precision (%CV) ≤ 25% and accuracy ± 25% of nominal values. Limit of detection was determined as the lowest standard with a signal-to-noise ratio of at least 3 and twice more than signal-to-noise ratio of single blanks. We use quality control samples (n ≥ 3) either prepared from pooled study samples or biological matrices with similar lipidome compositions in each run to capture the variabilities of all measurable lipid species. Herein, the within-run variabilities were determined using lipid concentrations measured in NIST 1950 plasma undiluted (n=6), 5x and 25x diluted (with 2% BSA, n=6). The experiment was repeated three consecutive times to evaluate between-run reproducibility. The concentrations of all quantified lipids were derived from the calibration curves in each run.

## Results

### Combining NPLC and HILIC to extend lipid class separation capacity

**Fig. 2:**
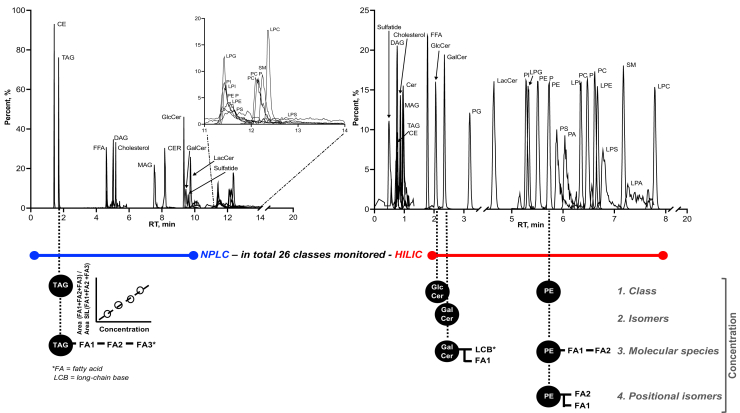
Chromatographic separation of the sMRM-NPLC/HILIC method providing different levels of quantitative information of measured lipids. Total Ion Chromatograms (TIC) of standards belonging to 26 different lipid classes monitored by NPLC and HILIC are shown respectively. The ion intensities of the measured standards were individually converted to percent distribution (y-axis) to better illustrate the chromatographic separation. The chromatograms were reconstructed in Prism 9.0. The taken chromatographic and MS acquisition strategy offer four levels of quantification, namely class, isomeric, molecular species, and positional isomers, illustrated with TAG, GlcCer, GalCer, and PE. GalCer, galactosyl-ceramide; GlcCer, glucosyl-ceramide; HILIC, hydrophilic interaction chromatography; NPLC, normal phase chromatography; QqQ, triple quadrupole.

We explored the application of multiplexing NPLC-HILIC to improve the separation of lipid classes than the uses of each method alone. Under the HILIC and NPLC chromatographic conditions (supplemental Table S5), a mixture of 26 lipid standards each representing a lipid class was tested on both chromatographic systems. NPLC provided baseline chromatographic separation of neutral lipids including cholesteryl esters (CE), cholesterol, glycerolipids, and free fatty acids and less hydrophilic lipids such as ceramides (Cer) as shown in Fig. 2. Baseline separation was achieved between CE and triacylglycerols (TAG), the two most lipophilic and abundant lipid classes, which is typically limited with HILIC. We obtained separation of diacylglycerols (DAG) 1,2 and 1,3-acyl positional isomers (supplemental Fig. S1A) on NPLC as reported elsewhere (22). GalCer and GlcCer were not baseline separated by NPLC being reported as hexosylceramide (HexCer). Although HexCer, Cer, lactosylceramide, and sulfatides displayed sharp and symmetrical peak shapes on NPLC, they eluted closely. NPLC offered limited separation to polar lipid classes such as the phospholipids. We decided to divert this part to waste, instead, monitor polar lipids in HILIC mode (Fig. 2). HILIC provided excellent separation for phospholipids classes, furthermore, baseline separation of isomeric classes such as GalCer and GlcCer, and *sn*-1 and *sn*-2 lysophospholipid isomers (23) (supplemental Fig. S1B). We note that individual lipid species within the same class did not overlap completely, rather eluted with very slightly different RT which could be predicted based on their carbon chain lengths and number of DBs (supplemental Fig. S4). It should be noted that additional lipid subclasses can readily be added into this platform beyond the studied herein. For example, we have observed excellent separation between isomeric phosphatidylglycerols (PGs) and bis(monoacylglycerol)phosphates as reported previously (24) in other data sets not included in this report. Lysophosphatidic acid was excluded due to peak tailing, which requires alternative chromatographic conditions for improvement.

### MS optimization for accurate lipid identification and quantitative performance

The high degree of heterogeneity across the molecular structures of glycerolipids, sterol lipids, sphingolipids, and phospholipids introduces distinct challenges to the development of an optimized detection method. We optimized and balanced single-run MS conditions to achieve lipid identification, detection sensitivity, and low in-source fragmentation (ISF). To build MRM transitions, we selected [M+H]^+^, [M+NH_4_]^+^, [M-H]^-^, and [M+OAc]^-^ precursor ions and monitored multiple structurally characteristic fragment ions of both ion modes (supplemental Table S2). RTs of lipid standards and ISs provide additional important information for identification.

Loss in MS detection sensitivity and ISF may occur to some lipids due to improper ion source settings. We observed marked improvements in sensitivity of neutral lipids and sphingolipids using lower ion source temperatures, whereas phospholipids detection was favored at relatively higher temperatures (supplemental Fig. S2). Neutral lipids were observed to be particularly sensitive to temperature-dependent ISF. For example, we observed a strong cholesteryl cation at *m/z* 369.3 at the RT for CE which resulted from neutral loss of ROOH of ammoniated CEs (25). We detected in-source losses of hexose, di-hexose, H_2_O, and sulfate (SO_3_) from HexCer, 2HexCer, and sulfatides (supplemental Fig. S3A2) which became more severe at a higher ion source temperature (e.g., 450°C vs. 300°C). ISF is inevitable and brings ambiguity into lipid annotation and quantification when chromatographic separation between classes is insufficient. As a case in point, close evaluation of the NPLC chromatographic data was required to discriminate closely eluting sulfatides, HexCer and dihexosylceramide (Hex2Cer) (Fig. 2). As shown in supplemental Fig. S3C, sulfatide d18:1d7/13:1 was observed to lose SO_3_ in source even at mild conditions to form an identical ion as GalCer d18:1d7/13:1. This type of ISF artifact can result in a pronounced contamination of the HexCer MRM transitions without sufficient chromatographic resolution. Thus, HILIC is more advantageous than NPLC in this situation due to better separation of these lipid species as shown in supplemental Fig. S3. We settled on 295°C and further softened other ion source parameters (e.g., declustering potential) for lipid classes prone to ISF. Cholesterol and other sterol lipids showed unsatisfactory sensitivity using ESI. A separate LC/MS analysis is required using positive atmospheric pressure chemical ionization detection monitoring [M-H_2_O+H]^+^ or [M-2H_2_O+H]^+^ (not shown).

### Multi-fragment MRM for deeper information

We chose to monitor multiple MS/MS fragments related to the headgroups, FAs, and long-chain bases of the same lipid species to provide additional structural information for peak identification (supplemental Table S2). Although targeted lipidomic methods often do not require multiple fragment ions for quantification, the rationale for adding this additional information in our strategy is two-fold. Firstly, using multiple fragment ions of the same molecular species reduces the false-positive rate (13, 26) of lipid identification. Mainly, it enables deeper lipidome information. Monitoring both acyl anions of diacylphospholipid species offers a potential to quantify alterations in positional isomers, that is, the FA distribution on the *sn*-1 and *sn*-2 positions of the glycerol backbone (27). To prove feasibility, we analyzed mixtures of isomeric-pure standards of phosphatidylcholine (PC) and phosphatidylethanolamine (PE) of 16:0/18:1 and 18:1/16:0 configurations at varying molar ratios and monitored both acyl anions FAs 16:0 and 18:1 by MRM. By plotting each FA peak area as a percent of both FAs, we obtained a linear response for both PC and PE (supplemental Fig. S5). Thus, from the linear regression lines, we could determine the amount of a particular positional isomer, for example, 30% FA 16:0 (Y-axis, supplemental Fig. S5A) translated to roughly 90% in the form of PE 16:0/18:1 and 10% as PE 18:1/16:0. The current approach is sufficient to estimate the relative change in the distribution of positional isomers, but absolute quantification of each positional isomeric species will require titration curves for each pair of isomeric-pure standards or by acquiring additional structural information by MS^3^ fragmentation where feasible (27). Another case is gaining FA composition for TAG. Here, we selected MRM transitions corresponding to [M+NH_4_]^+^ and [M-RCOOH+NH_4_]^+^ as precursor and daughter ions, respectively. We monitored the daughter ions resulting from the neutral losses of various fatty acids attached to three glycerol stereospecific positions to obtain fatty acids combinations associated with each TAG species. In this way, we can express TAGs not only as species (e.g., TAG 52:3) but also as proposed molecular species, for example, TAG 16:0_18:1_18:2.

The benefits of using several fragment ions per lipid species come at a cost as multiplexed lipid panels may quickly generate thousands of MRM transitions resulting in loss of precision. Although modern quadrupole mass spectrometers have adequate scanning speeds (e.g., 12,000 Da/sec for Sciex 6500 QqQ based on manufacturer) to run hundreds or thousands of MRM transitions with scheduling, a process is necessary in place to ensure the peak area precision for quantitation. We observed a trend of improvement in precision greater for lipids with comparatively sharper peaks such as CE (NPLC) and PE (HILIC) with reduced total transitions. Firstly, we prescreened lipid species against our MRM library to remove redundant MRM transitions and generated biological matrix-selected MRM’s as reported elsewhere (28, 29, 30). Then, we implemented precision checking (based on %RSD of the analyte over IS ratio of each lipid calculated from n ≥ 3 QC samples) as a routine method suitability test to guide the upper capacity of MRM transitions. Precision checking is especially important for complex lipid classes such as TAG that comprises of a wide range of individual molecular species eluting in a short time window. Preset precision criterion impacts significantly on the number of MRM transitions within an assay. Finally, isotopic overlaps are more of a concern for accuracy of quantification in TAG case, LICAR (21) isotope correction, or other tools must be applied to obtain accurate abundance.

Applying polarity switch between positive and negative ion modes within one MRM method provides higher throughput by monitoring more MRM transitions in one injection. However, our data indicate higher lower limits of quantitations and lower number of lipids can meet quantification criteria due to lowered scanning speed by two polarities as demonstrated using sulfatides in Table 1. The number of quantified PG were reduced from 84 to 36 and PCs from 109 to 78 using negative ion mode only and polarity switching, respectively (data not shown). Therefore, we apply polarity switching mainly for confirming lipid identities according to the guidelines by the Lipidomics Standards Initiative (11).

**Table 1:** Validation summary of the multiplexed LC-sMRM method

Lipid Class	LC/Ion Mode (±)	Calibration Standard	Internal Standard (Deuterium Labeled)	RT %CV (n=48)	R^2^^a^ Standard Curve	LOQ^b^ (μM)	No of Lipids Quantified^c^	Within-run Median %CV (n=6)	Between-runs Median %CV (n=6)
CE	NPLC/+	CE 18:1	CE 18:1d7	1.23	0.993	0.1075	22	25.3	6.6
TAG	NPLC/+	TAG 15:0_18:1_15:0	TAG 15:0_18:1d7_15:0	2.08	0.998	0.0087	260	13.8	9.3
DAG	NPLC/+	DAG 15:0_18:1	DAG 15:0_18:1d7	0.15	1	0.012	77 (1,2 DAG)	12	9.9
Cer	NPLC/+	Cer d18:1/16:0	Cer d18:1d7/16:0	0.17	0.999	0.0035	21	15.8	8.4
	NPLC/+	Cer d18:1/18:0	Cer d18:1d7/18:0	0.15	0.999	0.0035			
	NPLC/+	Cer d18:1/24:0	Cer d18:1d7/24:0	0.18	0.999	0.0088			
	NPLC/+	Cer d18:1/24:1	Cer d18:1d7/24:1	0.17	0.999	0.0044			
	NPLC/+	Cer d18:1/15:0	Cer d18:1d7/15:0	0.15	0.999	0.0067			
HexCer	NPLC/+	GlcCer d18:1/16:0	GlcCer 18:1d7/15:0	0.07	0.993	0.006	6	17.2	12
	NPLC/+	GalCer d18:1/12:0	GalCer 18:1d7/13:0	0.07	0.998	0.003			
Hex2Cer	NPLC/+	LacCer d18:1/16:0	LacCer 18:1d7/15:0	0.04	0.997	0.0625	5	22.4	7.3
LPI	HILIC/-	LPI 17:1	PG 15:0_18:1d7, LPI 17:1^d^	0.73	0.986	0.075	5	8	23.3
PG	HILIC/-	PG 15:0_18:1	PG 15:0_18:1d7	0.38	0.999	0.0185	84	4.3	16.3
PI	HILIC/-	PI 15:0_18:1	PI 15:0_18:1d7	0.57	0.998	0.0833	91	9.8	8.1
LPG	HILIC/-	LPG 17:1	PI 15:0_18:1d7, LPG 17:1^d^	0.63	0.994	0.1875	6 (0:0/FA)	4.9	11.6
PEP	HILIC/-	n/a	PE P-18:0/18:1d9	0.85	−	−	29	8.5	11.9
PE	HILIC/-	PE 15:0_18:1	PE 15:0_18:1d7	0.63	0.997	0.0055	91	9.7	10
PS	HILIC/-	PS 15:0_18:1	PS 15:0_18:1d7	2.03	0.998	0.3635	1	8.1	20.9
PCP	HILIC/-	n/a	PC P-18:0/18:1d9	0.66	−	−	29^e^	21	6.4
PC	HILIC/-	PC 15:0_18:1	PC 15:0_18:1d7	0.57	0.998	0.019	109	15.2	4.3
LPE	HILIC/-	LPE 18:1	LPE 18:1d7	0.29	0.997	0.03853	18 (0:0/FA)	6.5	11.6
LPS	HILIC/-	LPS 17:1	PS 15:0_18:1d7, LPS 17:1^d^	0.88	0.96	1.875	−	−	−
SM	HILIC/-	SM d18:1/18:1	SM d18:1d7/18:1	0.38	0.994	0.0384	21	17.9	19.8
LPC	HILIC/-	LPC 18:0	LPC 18:1d7	0.49	0.999	0.0134	18 (0:0/FA)	10.4	3.7
FFA^g^	HILIC/-	n/a	FFA 18:0d3	1.6	−	−	5^f^	14	−
SHexCer^g^	HILIC/+	SHexCer d18:1/17:0	SHexCer d18:1d7/13:0	2.04 (n=12)	0.999	0.00625	9	11.9	n/a
	HILIC/-	SHexCer d18:1/17:0	SHexCer d18:1d7/13:0	1.7 (n=12)	0.997	0.025	3	23	n/a
MAG^g^	HILIC/+	MAG 18:1	MAG 18:1d7	1.7 (n=12)	0.992	0.13	6	15.1	n/a

a linear regression, 1/x weighted.

b defined as within 25% accuracy.

c within-run %CV <25%, in NIST-SRM-1950.

d not available at the time of between-run validation.

e identified as PC-O species.

f 16:1, 18:1, 18:2, 20:4 and 22:6 acceptable; other FFAs rejected due to MRM interferences.

g Within-run performance in HILIC ± ion polarity switching.

### Assay validation and sample analyses

The chosen lipid extraction method provided acceptable and reproducible extraction recoveries for most of the lipid classes monitored, ranging between 69–115%, with the exception of lysophosphatidylinositol, lysophosphatidylserine, and lysophosphatidylglycerol species that were found to exhibit lower recoveries similar to levels previously reported (31) (supplemental Table S6). We obtained linear response for most of the lipid classes with correlation coefficient (*R*^2^) above 0.99 (Table 1). LOQ for each class were determined in good alignments with previous studies (32, 33). Moreover, RTs across all lipid classes were stable and within acceptable %RSD values of 0.04–2.5%.

**Fig. 3:**
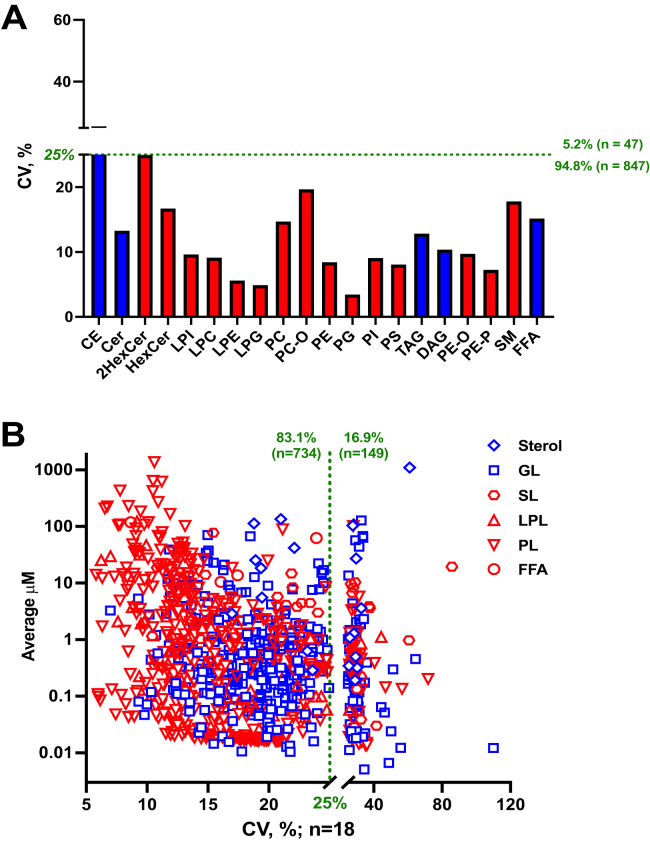
Within- and between-assay precision of measured lipid signals in 1950 NIST plasma samples. The % CVs for each lipid signal was calculated from the analysis of six constitutive individual samples (n=6) to determine the within-assay precision (A). Similarly, analysis of the six QCs was repeated over 3-days to determine the between-assay precision (n=18) (B). In within-assay, almost 95% of the signals out of the 894 recorded lipid signals fall inside the 25% acceptable threshold. The % CVs of each class, represented as the mean of all signals belonging to selective classes, are below 25% irrespective of analyzed in NPLC (blue) or HILIC (red). The tolerant coverage decreases to 83% when the analysis is repeated over 3 days. Still, 734 lipid signals do not deviate more than 25%. Presenting the lipid signals based on their lipid category shows lowest variation in phospholipids and highest in glycerolipids. Lipid categories sterol (rhombus) and glycerolipids (GL, square) monitored in NPLC are in blue, and sphingolipids (SL, hexagon), lysophospholipids (LPL, triangle), phospholipids (PL, inverted triangle), and free fatty acids (FFA, circle) monitored in HILIC are in red. HILIC, hydrophilic interaction chromatography; NPLC, normal phase chromatography.

We evaluated within-run and between-run reproducibility for NIST human plasma analyses utilizing an MRM panel corresponding to human plasma lipid composition (29, 30, 32). We chose to monitor apolar lipids from CE to lactosylceramide in NPLC positive ion mode, whereas all other, more polar classes in HILIC negative mode. We also show sulfatide and monoacylglycerol results in polarity switching mode from a separate experiment (Fig. 2 and Table 1). We were able to report close to 900 lipid ions, distributed among the lipid classes according to Table 1. Using a median within-run %CV (n=6) < 25% as the threshold, all lipid classes passed acceptance criteria with the exception of CE (25.3%). For each individual lipid, we identified 94.8% (equivalent to 843 number of) lipids with %CV less than 25% within-run, whereas the rest 5.2% (equivalent to 46 number of) lipids did not exceed 60% (Fig. 3A). The percentage of %CV >25% increased to 17% from between-run evaluation (n=6 each run and 3 independent runs), corresponding to 149 lipid ions (Table 1 and Fig. 3B). The between-run results confirmed that 729 plasma lipid species could be quantified with a variability of less than 25%. It is worth noting most of the 149 lipids resided in a %CV between 25–40%. We observed 20–16% improvement in between-run precision when signals from two MRM transitions representing both acyl anions of phospholipids were summed. This resulted in 84% of phospholipids meeting the 25% cutoff instead of 75% for single MRMs (data not shown).

Our multiple MRM per lipid approach correlates well quantitatively to previous studies by Bowden *et al.* (28) and Ghorasaini *et al.* (34) (supplemental Fig. S7A). However, we note that these studies quantify lipids at sum composition level, whereas our method provides quantification of molecular lipid species a deeper coverage of studied lipidomes. For instance, we could quantify three molecular species with the sum compositions PC 36:2 and four different molecular species for PC 38:4 (supplemental Fig. S7B). Importantly, when we sum up the concentrations of the individual molecular species, we reach close consensus with the above-mentioned reports using sum compositions. This further verifies the validity of the quantitative accuracy of our method in addition to gaining a deeper level of lipidome information.

To address the impact of matrix effect and dilution integrity, a requirement in FDA industry guidance for endogenous analytes, we evaluated sample dilution and found satisfactory results by plotting the *R*^2^ of MS responses from high-abundance lipids in undiluted, 5x and 25x diluted NIST-SRM-1950 plasma (supplemental Fig. S6A). We found that the upper limits of quantitation (ULOQs) (defined by calibration curves, supplemental Table S3) could be extended further for most lipid classes because the peak area ratios (analyte over IS) of the lipids exceeding the ULOQs were still linear with those of 5x and 25x diluted. As shown in supplemental Fig. S6B using sphingomyelin (SM) d18:1/16:0 as an example, the SM ULOQ could be extended to 2x higher than the current 15.3 μM. Therefore, we included QC analyses of serial dilutions of 1950 NIST plasma (or pooled study samples) as a routine strategy for ULOQ extension.

Although we found standard curves (slopes, lower limits of quantitation, ULOQ) had good reproducibility over time in general, we did observe drift in the slopes of the calibration curves, most frequently for sphingolipid classes during analysis of large batch of samples. The standard curves also varied on different mass spectrometers. Signal drifting over time and batch-to-batch analytical heterogenicity is common in LC/MS-based omics methods and several correction strategies have been developed (35, 36). We found that inserting multiple standard curves (one curve minimum per 96-well plate) and QC samples (n=6 per 96-well plate in general, or one QC every 20–30 samples) evenly among study samples was a simple and effective way to correct signal drifting. When we derived concentrations from the most adjacent standard curve, as exemplified in supplemental Table S7, the precision (%CV) of SM d18:1/18:1 is improved from 25% to 12.6% in a multi-day analysis. We demonstrate here an effective within-run solution that is alternate to other approaches (32, 37). This approach also minimizes data discrepancy when analyses are performed on different LC-MS systems.

### Identification of alterations in the plasma lipidome following GCS inhibition

**Fig. 4:**
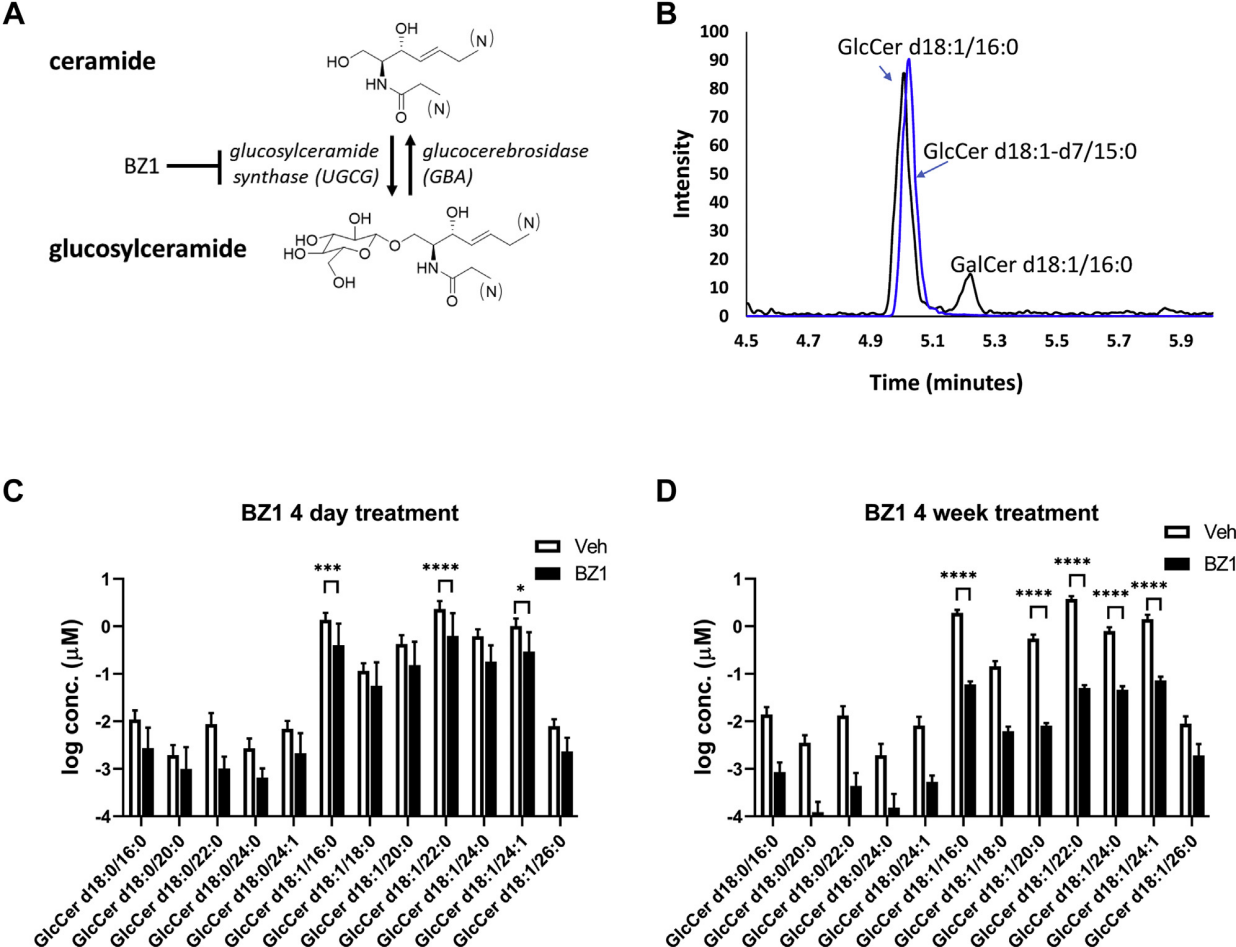
Chronic BZ1 treatment lowers circulating glucosylceramides. A: Circulating levels of glucosylceramide are regulated through the balance of synthesis and catabolism via glucosylceramide synthase, encoded by the gene UGCG, and cytosolic and lysosomal glucocerebrosidases encoded by GBA genes. BZ1 is a potent glucosylceramide inhibitor. B: Baseline separation of plasma glucosylceramide and galactosylceramide isomeric species is achieved using HILIC chromatography and MS acquisition in positive mode within the global assay method. C: Plasma glucosylceramides are reduced in D409V mice following four days of BZ1 treatment compared to control (veh) (N=7). D: BZ1 treatment for four weeks reduces the major glucosylceramide species by approximately 80%–90% of concentrations observed in matched vehicle-treated mice (N=5). BZ1, benzoxazole; HILIC, hydrophilic interaction chromatography. Error bars span standard deviation values; asterisks denote statistical significance as (∗) *P* < 0.05, (∗∗∗) *P* < 0.001, (∗∗∗∗) *P* < 0.0001 using multiple *t*-tests.

We quantified plasma lipids in mice treated with BZ1, a potent inhibitor of GCS, as a demonstration of utility of our assay platform using a well-defined model of pharmacology. GCS inhibition reduces circulating glucosylceramide levels (20), but effects on the lipidome beyond glycosphingolipid metabolism are not well understood. The gene GBA1 encodes glucocerebrosidase, a lysosomal enzyme that hydrolyzes the glucosylceramides to Cers (Fig. 4A). Mutations in GBA have been identified as a genetic risk factor for Parkinson’s disease (38, 39), and mice that are heterozygous for the D409V GBA mutation recapitulate glycosphingolipid phenotypes of GBA-related Parkinson’s disease (40, 41). We measured plasma lipids from D409V GBA heterozygous mice fed either control or BZ1-formulated chow for four days or four weeks to reduce glucosylceramide synthesis rates. BZ1 plasma exposure levels were sufficient to maintain GCS target engagement at four days and four weeks roughly 2.0-fold and 4.6-fold the GCS enzyme IC50 values, respectively.

### Mice treated with BZ1 exhibited marked reductions in plasma glucosylceramides

The chromatograms in Fig. 4B were obtained by HILIC and demonstrate baseline separation of endogenous HexCer d18:1/16:0. The plasma concentrations of GlcCer species measured in vehicle-treated mice replicated previously reported concentration distributions (42). Reduction of GlcCer chain length variants verified GCS target engagement by BZ1 and differences in GlcCer reductions between 4 days- and 4 weeks-treated mice exhibited a dose-response like effect (Fig. 4C, D).

### Shifts in the plasma lipidome extend beyond the sphingolipids

**Fig. 5:**
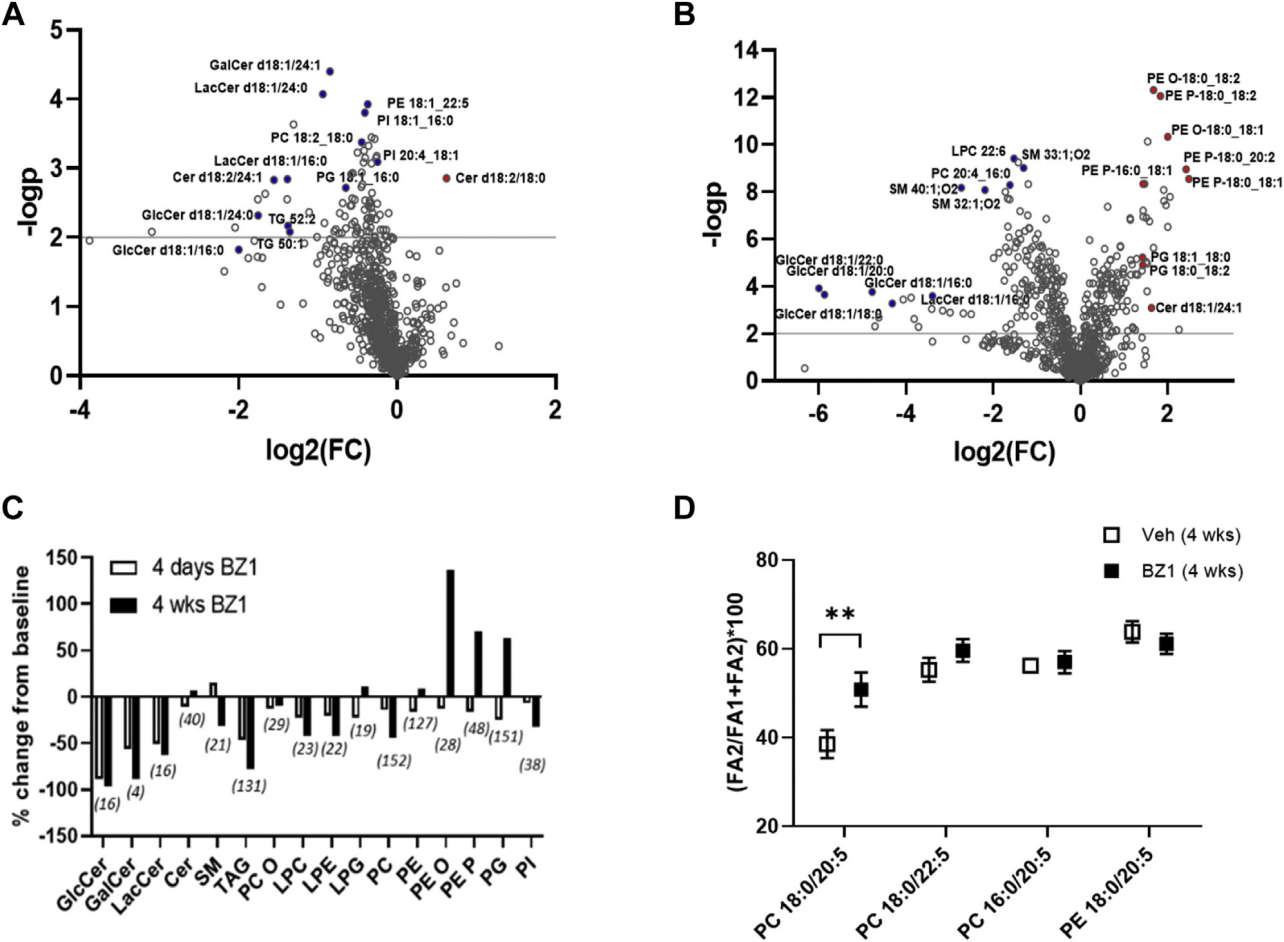
Secondary alterations in the plasma lipidome correlated with chronic BZ1 inhibition of GCS. Volcano plots of targeted plasma lipids labeled selectively following feeding of BZ1 diet for (A) four days and (B) four weeks identify treatment-dependent pharmacodynamic effects within the plasma lipidome beyond reduced glucosylceramide levels. C: Percent changes across lipid classes calculated as the sum of individual species and normalized to vehicle-treated controls provide a high-level descriptor of BZ1 treatment-dependent effects on plasma lipids. Number of detected lipid molecular species are in parentheses. D: Shifts in the distribution of fatty acyl positional isomers of PC18:0/20:5 are observed in BZ1-treated animals. BZ1, benzoxazole; GCS, glucosylceramide synthase. Error bars span standard deviation values; asterisks denote statistical significance as (∗∗) *P* < 0.01 using 2-way ANOVA.

BZ1-treated mice exhibited broad alterations in circulating lipids compared to vehicle-treated controls. The volcano plots in Fig. 5 summarize relative changes in 862 detected plasma lipid species from mice treated with BZ1 for four days (Fig. 5A) and four weeks (Fig. 5B) compared to matched vehicle controls. As expected, GlcCer's, our positive control for pharmacodynamic effect, are among the analytes exhibiting the greatest reductions in both 4-days and 4-weeks treatment groups. However, the alterations in the plasma lipidome are beyond GlcCer's, affecting many various species. There are marked alterations across lipid species such as distinct glycerolipids and phospholipids that can be considered relatively distant from glucosylceramide synthesis. Figure 5C plots the percent change across lipid classes derived from the sum of lipid species relative to control groups. BZ treatment is correlated with aggregate reductions in TAGs, DAGs, phosphatidylinositols, and PCs in both treatment groups. Additionally, marked elevations in circulating alkyl PE (PE O), plasmalogen PE and PG classes are observed in the 4-weeks BZ1 treatment groups. The extended effects of chronic GCS inhibition observed in the plasma lipidome are an indication of off-target activity of the BZ1 molecule. Being used as a tool for drug development, this lipidomic platform enables rapid assessment of target selectivity and can guide compound differentiation in preclinical studies.

Deeper structural information acquired from both acyl anions belonging to diacylphospholipid species revealed treatment-dependent shifts in the relative distribution of positional isomers. We identified changes in selective species such as PC 18:0_20:5 (Fig. 5D). We observed approximately 85% in the form of PC 20:5/18:0 (relative to total 18:0/20:5 and 20:5/18:0) from vehicle-treated mice at 4 weeks. In BZ1-treated mice, this ratio shifted to approximately 50%, estimated from our titration curves using PC16:0_18:1 (supplemental Fig. S5B). These data indicated that GCS inhibition by BZ1 correlated the shift in this pair of positional isomers where PUFAs (FA 20:5) translocating to the *sn*-2 position of the glycerol backbone, which is known to be the predominant position for PUFA. We note that these were estimates due to the possible response deviations of PC 18:0_20:5 in correspondence to PC 16:0_18:1 that was used in our titration experiments (supplemental Fig. S5B). To our knowledge, this is the first report in which shifts in lipid positional isomers have been identified.

**Fig. 6:**
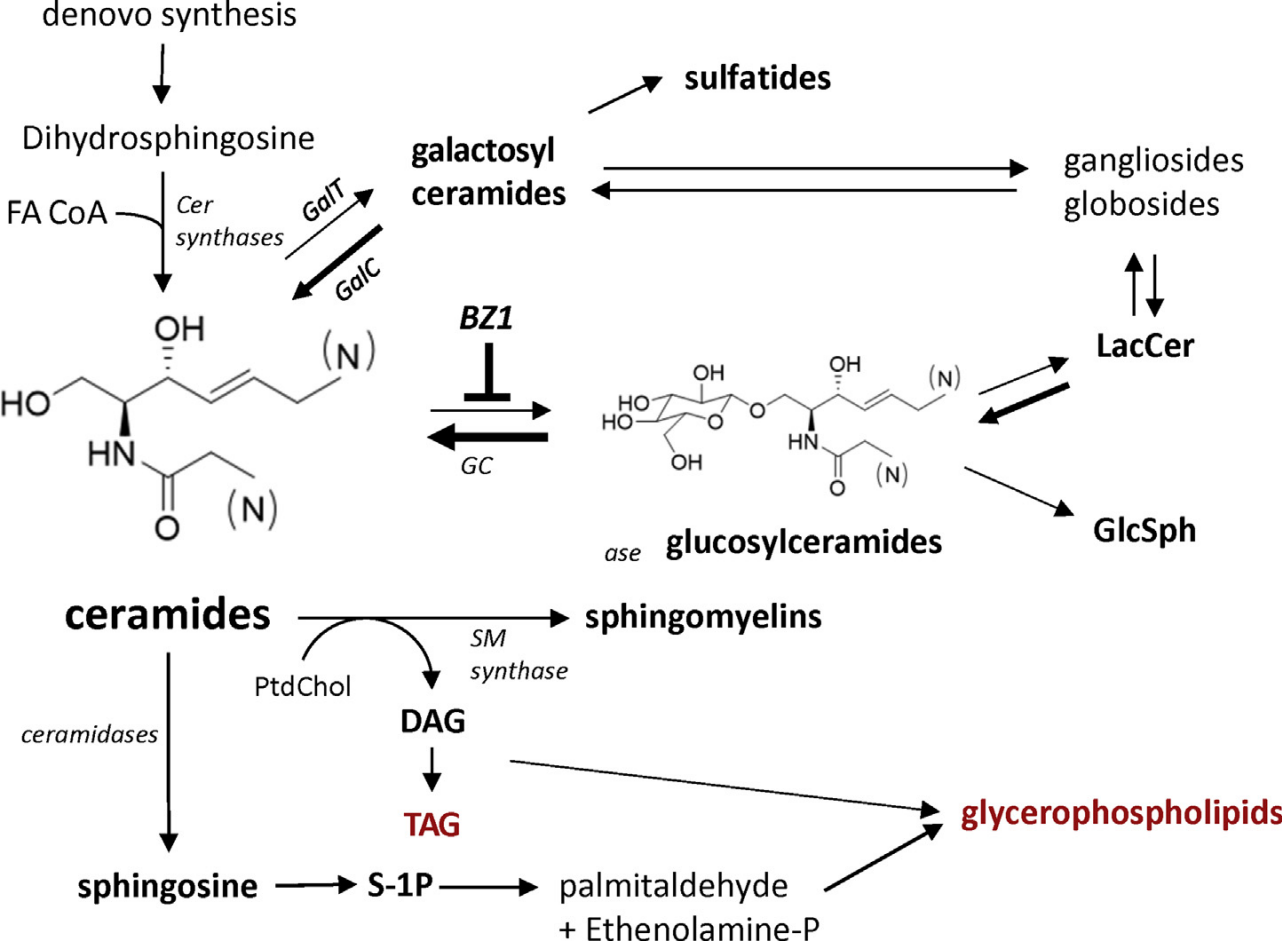
Schematic of metabolic pathways linking GCS inhibition to secondary alterations in glycerolipids and glycerophospholipids. GCS, glucosylceramide synthase.

The broad effects observed in the lipidome of BZ1-treated mice may be a result of the interdependent nature of the lipid metabolic network. Perturbations in a single node may alter equilibria of many downstream metabolites as postulated by Fuller and Futerman (43). Here, chronic inhibition of GlcCer synthase in the liver and other organs resulted in robust, long-term reductions in GlcCer's and related glycosphingolipids as represented in Fig. 6. Rather than accumulated, Cer substrate was metabolized through a number of pathways including deacylation to sphingosine as well as conversion to SM concomitant with DAG production from PC. DAG metabolism could be viewed as another major, interconnected hub within lipid metabolic pathways acting as a starting substrate leading to alterations across glycerophospholipid classes observed in our BZ1 rodent study. We overlayed the potential circulating metabolic endpoints originating from DAG in BZ1-treated mice at 4-days versus 4-weeks in supplemental Fig. S8. These data-emerged trends showed continued reductions in PI’s, SM’s, and PC’s, mixed effects across PE structural variants, and increased flux toward production of PG and plasmalogen PE species during the course of chronic GCS inhibition. Thus, long-term inhibition of GCS inhibition resulted in alterations in lipid metabolic pathways that manifest as alterations through large swaths of the lipidome.

## Discussion

An accurate accounting of the lipidome in cells, tissues, and fluids is critical to understanding the links between lipid dysregulations and diseases. Lipidomic analyses should seek to provide quantitative and reproducible measures of broad lipid species present in lipidomes with sufficient depth in structural information. Assay accuracy, precision, and linear dynamic range often fall short in lipidomics which is reflected in the wide issue of data irreproducibility (13). Although standardization within the field is underway by the Lipidomics Standards Initiative (11), common problems of improper annotation of lipid species, misidentifications, and over-reporting among others will continue until minimal guidelines have been published and implemented. To address these issues, we incorporated quality criteria guidance recommendations from the FDA BA method validation for industry (15) into our data sets and implemented isotope correction to achieve accurate quantification of our targeted lipid analytes. We demonstrate using selected lipid classes with appropriate lipid standards available and show upbuilding potential of this platform.

Here, we describe a multiplexed NPLC-HILIC QqQ MRM based lipidomics method to quantify over twenty lipid classes and subclasses. Utilizing the separation capability enhanced by dual chromatographic method and fast scanning QqQ MRM detection at positive and negative ionization modes, we enable lipidome profiling with adequate structural specificity for biological discovery and quantitative reproducibility with relatively fast turnaround time from analyses to final lipid concentrations. In addition, we describe processes to ensure reliable lipidomic quantification using triple quadrupole MRM detection and a quality control process for sample analysis. A common concern of MRM lipidomics is insufficient data points across peaks. We observed average data points across peaks varied from 7 to 51 for the deuterated lipid ISs of each lipid class yielding satisfactory results with this method. This observation agrees with previous reports (44, 45).

As a case study, we report pharmacodynamic alterations in the plasma lipidome following inhibition of GCS in a well-defined mouse GBA deficiency model. Our data replicate the expected magnitude of reductions of glucocerebroside-specific lipid molecular species and, more importantly, reveal novel alterations in the plasma lipidome beyond the proximal GBA-related sphingolipid species. Although additional experiments are needed to resolve the biology, the current experiments identify expanded lipidomic phenotypes that altered with GCS inhibition thus providing deeper insights into the underlying metabolic mechanisms as well as identification of new lipid biomarkers relating GCS inhibition.

We recognize ultrahigh-performance supercritical fluid chromatography (UHPSFC) offers separation of lipid classes within comparatively short run times (32, 37, 46). However, the utility of UHPSFC remains uncommon among large-scale applications, and UHPSFC methods offering simultaneous separation of most lipophilic lipid classes (e.g., TAG and CE) and polar isomeric lipids (e.g., GlcCer and GalCer) in a single run have not been described. We note here addition of ion mobility to MS as an orthogonal dimension empowers lipid separation, especially for lipid isomers (47, 48). Ion mobility-based approaches hold great promise for use in scalable applications with quick turnaround times as the hardware technology and data processing workflows continue to evolve.

## Data availability

This article contains supplemental data. All data supporting this study are included in the article and the supplemental data.

## Supplemental data

This article contains supplemental data (49).

## Conflicts of interest

The authors declare that they have no competing interests on every aspect of the work.
