# Supplementary material for: Validation of a multiplexed and targeted lipidomics assay for accurate quantification of lipidomes
Source: J Lipid Res. 2022 Apr 27;63(6):100218. doi: 10.1016/j.jlr.2022.100218 (PMC9168725; doi:10.1016/j.jlr.2022.100218)
Supplement: Lipidomics_Supporting_11April2022_clean.docx [file mmc1.docx]

SUPPORTING INFORMATION

**Validation of a multiplexed and targeted lipidomics assay for accurate quantification of lipidomes**

Nanyan Rena Zhang*^1^, Nathan G. Hatcher*^2^, Kim Ekroos^3^, Komal Kedia^1^, Monika Kandebo^2^, Jacob N. Marcus^2^, Sean M. Smith^2^, Kevin P. Bateman^1^, Daniel S. Spellman^1^

^1^Department of Discovery, Preclinical and Translational Medicine, Merck & Co., Inc., West Point, PA 19486 USA

^2^Department of Neuroscience, Merck & Co., Inc., West Point, PA 19486 USA

^3^Lipidomics Consulting Ltd, Irisviksvägen 31D, Esbo, Finland

**Corresponding author:* [*rena_zhang@merck.com*](mailto:rena_zhang@merck.com)*,* [*Nathan_hatcher@merck.com*](mailto:Nathan_hatcher@merck.com)*;*

**SUPPORTING INFORMATION TABLE OF CONTENTS**

Supplemental Table S1 page 2

Supplemental Table S2 page 2

Supplemental Table S3 page 4

Supplemental Table S4 page 4

Supplemental Table S5 page 5

Supplemental Table S6 page 5

Supplemental Table S7 page 7

Supplemental Figure S1 page 8

Supplemental Figure S2 page 9

Supplemental Figure S3 page 10

Supplemental Figure S4 page 11

Supplemental Figure S5 page 12

Supplemental Figure S6 page 13

Supplemental Figure S7 page 14

Supplemental Figure S8 page 15-16

References page 17

TAG 15:0_18:1, Rt=1.3

CE 18:1, Rt=1.3

**Supplemental Table S1.** Summary of lipid standards tested in Method Validation and sample analysis.

| **Lipid** | **Standard** | **Deuterated Standard** |
| --- | --- | --- |
| Phosphatidylcholine (PC) | PC 15:0_18:1 | PC 15:0_18:1d7 |
| Lysophosphatidylcholine (LPC) | LPC 18:1 | LPC 18:1d7 |
| Plasmenyl Phosphatidylcholine (PC P) | PC P 18:0/18:1 | PC P-18:1/18:1d9 |
| Phosphatidylethanolamine (PE) | PE 15:0_18:1 | PE 15:0_18:1d7 |
| Lysophosphatidylethanolamine (LPE) | LPE 18:1 | LPE 18:1d7 |
| Plasmenyl Phosphatidylethanolamine (PE P) | PE P 18:0/18:1 | PE P 18:1/18:1d9 |
| Phosphatidylglycerol (PG) | PG 15:0_18:1 | PG 15:0_18:1d7 |
| Lysophosphatidylglycerol (PG) | LPG 17:1 | LPG 17:0 d5 |
| Phosphatidylinositol (PI) | PI 15:0_18:1 | PI 15:0_18:1d7 |
| Lysophosphatidylinositol (LPI) | LPI 17:1 | LPI 17:0 d5 |
| Phosphatidylserine (PS) | PS 15:0_18:1 | PS 15:0_18:1d7 |
| Lysophosphatidylserine (LPS) | LPS 17:1 | LPS 17:0 d5 |
| Phosphatidic acid (PA) | PA 15:0_18:1 | PA 15:0_18:1d7 |
| Lysophosphatidic acid (LPA) | LPA 16:0 |  |
|  | LPA 17:0 |  |
| Triacylglycerols (TAG) | TAG 15:0_18:1_15:0 | TAG 15:0_18:1d7_15:0 |
| Diacylglycerols (DAG) | DAG 15:0_18:1 | DAG 15:0_18:1d7 |
| Monoacylglycerols (MAG) | MAG 18:1 | MAG 18:1d7 |
| Cholesteryl Ester (CE) | CE 18:1 | CE 18:1d7 |
| Cholesterol | Cholesterol | Cholesterol-d7 |
| Sphingomyelins (SM) | SM d18:1/18:1 | SM d18:1d7/18:1 |
| Ceramide (Cer) | Cer d18:1/15:0 | Cer d18:1d7/15:0 |
|  | Cer d18:1/16:0 | Cer 18:1d7/16:0 |
|  | Cer d18:1/18:0 | Cer d18:1d7/18:0 |
|  | Cer d18:1/24:0 | Cer d18:1d7/24:0 |
|  | Cer d18:1/24:1 | Cer d18:1d7/24:1 |
| Galactosylceramide (GalCer) | GalCer d18:1/12:0 | GalCer d18:1d7/13:0 |
| Glucosylceramide (GlcCer) | GlcCer d18:1/16:0 | GlcCer d18:1d7/15:0 |
| Lactosylceramide (LacCer) | LacCer d18:1/16:0 | LacCer d18:1d7/15:0 |
| Sulfatide (SHexCer) | SHexCer d18:1/17:0 | SHexCer d18:1d7/13:0 |
|  | SHexCer d18:1/24:1 |  |
| Free fatty acid (FFA) | FFA 18:2 | FFA 18:0d3 |
| *sn*-1 and *sn*-2 isomer pure phospholipids | 18:1(n9)/16:0 PE ISOPUR | PE 15:0_18:1d7 |
|  | 16:0/18:1(n9) PE ISOPUR |  |
|  | 16:0/18:1(n10) PC ISOPUR | PC 15:0_18:1d7 |
|  | 18:1(n10)/16:0 PC ISOPUR |  |

**Supplemental Table S2.** Lipid-class characteristic ion-based MRM transitions selected for lipid identification, using 18:0 and 18:1 fatty acid composition as representatives.

| Lipid class | NPLC | HILIC | Annotation | ESI mode | Precursor *m/z* | Precursor name | MS2 fragments^a^ | CE (eV) |
| --- | --- | --- | --- | --- | --- | --- | --- | --- |
| CE | √ |  | CE 18:1 | + | 668.6 | [M+NH_4_]^+^ | 369.4 | 30 |
| TAG | √ |  | TAG (44-58):(0-9)-FA18:0 | + | 850.8 | [M+NH_4_]^+^ | 549.5 | 38 |
| Cholesterol | √ |  | Cholesterol | + | 369 | [M+H-H_2_O]^+^ | 161, 147 | 45 |
| DAG | √ |  | DAG 18:0_18:1 | + | 640.4 | [M+NH_4_]^+^ | 341.2 | 25 |
|  |  |  |  | + | 640.4 | [M+NH4]^+^ | 339.2 | 45 |
| MAG | √ |  | MAG 18:1 | + | 357.3 | [M+H]^+^ | 247.2 | 25 |
|  |  |  |  | + | 374 | [M+NH_4_]^+^ | 265.2 | 25 |
| FFA | √ | √ | FFA 18:0 | - | 283.2 | [M-H]^-^ | 283.2 | -5 |
| Cer | √ | √ | Cer d18:1/18:0 | + | 566.7 | [M+H]^+^ | 264.2 | 40 |
|  |  |  | Cer d18:0/18:1 | + | 566.8 | [M+H]^+^ | 266.2 | 40 |
|  |  |  | Cer d16:1/18:0 | + | 538.5 | [M+H]^+^ | 236.2 | 40 |
|  |  |  | Cer d18:2/18:0 | + | 564.5 | [M+H]^+^ | 262.2 | 40 |
| GlcCer/GalCer^b^ | √ | √ | HexCer d18:1/18:0 | + | 728.6 | [M+H]^+^ | 264.2 | 45 |
|  |  |  | HexCer d18:0/18:0 | + | 730.7 | [M+H]^+^ | 266.2 | 45 |
| 2HexCer | √ | √ | 2HexCer d18:1/18:0 | + | 890.7 | [M+H]^+^ | 264.2 | 45 |
|  |  |  | 2HexCer d18:0/18:0 | + | 892.7 | [M+H]^+^ | 266.2 | 45 |
| PG |  | √ | PG 18:0_18:1, PG 36:1 | - | 775.5 | [M-H]^-^ | 283.3 | -50 |
|  |  |  |  | - | 775.5 | [M-H]^-^ | 281.2 | -50 |
| PI |  | √ | PI 18:0_18:1, PI 36:1 | - | 863.6 | [M-H]^-^ | 283.3 | -50 |
|  |  |  |  | - | 863.6 | [M-H]^-^ | 281.2 | -50 |
| LPG |  | √ | LPG 18:1 | - | 509.3 | [M-H]^-^ | 281.2 | -50 |
| PA |  | √ | PA 18:0_18:1, PA 36:1 | - | 701.5 | [M-H]^-^ | 283.3 | -50 |
|  |  |  |  | - | 701.5 | [M-H]^-^ | 281.2 | -50 |
|  |  |  |  | - | 701.5 | [M-H]^-^ | 153.0 | -50 |
| PE |  | √ | PE 18:1_18:0, PE 36:1 | - | 744.6 | [M-H]^-^ | 281.2 | -50 |
|  |  |  | PE 18:0_18:1 | - | 744.6 | [M-H]^-^ | 283.3 | -50 |
| PE O |  | √ | PE O-18:0/18:1 | - | 730.6 | [M-H]^-^ | 281.2 | -50 |
| PE P |  | √ | PE P-18:1/18:0 | - | 728.6 | [M-H]^-^ | 281.2 | -50 |
| PS |  | √ | PS 18:0_18:1 | - | 788.5 | [M-H]^-^ | 283.3 | -40 |
|  |  |  | PS 18:1_18:0 | - | 788.5 | [M-H]^-^ | 281.2 | -40 |
| LPI |  | √ | LPI 18:1 | - | 597.3 | [M-H]^-^ | 281.2 | -50 |
|  |  |  |  | - | 597.3 | [M-H]^-^ | 153.0 | -50 |
| PC O |  | √ | PC O-36:1 | + | 774.7 | [M+H]^+^ | 184.1 | 50 |
|  |  |  | PC O-18:0/18:1, PC O-36:1 | - | 832.7 | [M+OAc]^-^ | 281.2 | -50 |
| LPE |  | √ | LPE 18:1 | - | 478.3 | [M-H]^-^ | 281.2 | -40 |
| LPS |  | √ | LPS 18:1 | - | 522.3 | [M-H]^-^ | 153.0 | -50 |
|  |  |  |  | - | 522.3 | [M-H]^-^ | 281.2 | -50 |
| PC |  | √ | PC 18:0_18:1, PC 36:1 | - | 846.6 | [M+OAc]^-^ | 283.3 | -50 |
|  |  |  |  | - | 846.6 | [M+OAc]^-^ | 281.2 | -50 |
|  |  |  |  | + | 788.7 | [M+H]^+^ | 184.1 | 70 |
| LPA |  | √ | LPA 18:1 | - | 417.2 | [M-H]^-^ | 153.0 | -50 |
|  |  |  |  | - | 417.2 | [M-H]^-^ | 281.2 | -50 |
| ShexCer | √ | √ | SHexCer d18:1/18:0 | + | 808.6 | [M+H]^+^ | 264.2 | 50 |
|  |  |  | SHexCer d36:1 | - | 806.5 | [M-H]^-^ | 97.0 | -75 |
| SM |  | √ | SM 36:1 | + | 731.6 | [M+H]^+^ | 184.1 | 70 |
|  |  |  |  | - | 789.6 | [M+OAc]^-^ | 168.1 | 50 |
| LPC |  | √ | LPC 18:1 | + | 522.5 | [M+H]^+^ | 184.1 | 70 |
|  |  |  |  | - | 580.4 | [M+OAc]^-^ | 580.4 | 50 |

^a^ see Ståhlman et al (1).

^b^ HexCer applies to NPLC whereas GlcCer and GalCer for HILIC.

**Supplemental Table S3.** Concentration (μM) for lipid calibrators used for **Method Validation**.

| Standard | std1 | std2 | std3 | std4 | std5 | std6 | std7 | std8 | std9 | std10 |
| --- | --- | --- | --- | --- | --- | --- | --- | --- | --- | --- |
| CE 18:1 | 0.0054 | 0.0107 | 0.022 | 0.043 | 0.108 | 0.430 | 1.07 | 4.30 | 8.60 | 17.19 |
| TAG 15:0_18:1_15:0 | 0.0043 | 0.0087 | 0.017 | 0.035 | 0.087 | 0.348 | 0.87 | 3.48 | 6.95 | 13.90 |
| DAG 15:0_18:1 | 0.0060 | 0.0120 | 0.024 | 0.048 | 0.120 | 0.482 | 1.20 | 4.82 | 9.63 | 19.27 |
| Cer d18:1/15:0 | 0.0067 | 0.0134 | 0.027 | 0.053 | 0.134 | 0.534 | 1.34 | 5.34 | 10.68 | 21.37 |
| Cer d18:1/16:0 | 0.0035 | 0.0070 | 0.014 | 0.028 | 0.070 | 0.281 | 0.70 | 2.81 | 5.63 | 11.25 |
| Cer d18:1/18:0 | 0.0018 | 0.0035 | 0.007 | 0.014 | 0.035 | 0.141 | 0.35 | 1.41 | 2.82 | 5.64 |
| Cer d18:1/24:0 | 0.0088 | 0.0176 | 0.035 | 0.070 | 0.176 | 0.704 | 1.76 | 7.04 | 14.08 | 28.17 |
| Cer d18:1/24:1 | 0.0044 | 0.0088 | 0.018 | 0.035 | 0.088 | 0.352 | 0.88 | 3.52 | 7.04 | 14.07 |
| GlcCer d18:1/16:0 | 0.0016 | 0.0031 | 0.006 | 0.013 | 0.025 | 0.063 | 0.25 | 0.63 | 1.25 | 2.50 |
| GalCer d18:1/12:0 | 0.0016 | 0.0031 | 0.006 | 0.013 | 0.025 | 0.063 | 0.25 | 0.63 | 1.25 | 2.50 |
| LacCer d18:1/16:0 | 0.0016 | 0.0031 | 0.006 | 0.013 | 0.025 | 0.063 | 0.25 | 0.63 | 1.25 | 2.50 |
| LPI 17:1 | 0.0094 | 0.0188 | 0.038 | 0.075 | 0.188 | 0.750 | 1.88 | 7.50 | 15.00 | 30.00 |
| PG 15:0_18:1 | 0.0046 | 0.0092 | 0.018 | 0.037 | 0.092 | 0.370 | 0.92 | 3.70 | 7.39 | 14.79 |
| PI 15:0_18:1 | 0.0042 | 0.0083 | 0.017 | 0.033 | 0.083 | 0.333 | 0.83 | 3.33 | 6.66 | 13.33 |
| LPG 17:1 | 0.0090 | 0.0190 | 0.038 | 0.075 | 0.188 | 0.750 | 1.88 | 7.50 | 15.00 | 30.00 |
| PE 15:0_18:1 | 0.0050 | 0.0099 | 0.020 | 0.040 | 0.099 | 0.398 | 0.99 | 3.98 | 7.95 | 15.90 |
| PS 15:0_18:1 | 0.0045 | 0.0091 | 0.018 | 0.036 | 0.091 | 0.363 | 0.91 | 3.63 | 7.27 | 14.54 |
| PC 15:0_18:1 | 0.0047 | 0.0094 | 0.019 | 0.038 | 0.095 | 0.375 | 0.94 | 3.75 | 7.50 | 15.00 |
| LPC 18:1 | 0.0067 | 0.0134 | 0.027 | 0.054 | 0.134 | 0.536 | 1.34 | 5.36 | 10.73 | 21.46 |
| LPE 18:1 | 0.0073 | 0.0146 | 0.029 | 0.058 | 0.146 | 0.584 | 1.46 | 5.84 | 11.67 | 23.34 |
| LPS 17:1 | 0.0094 | 0.0188 | 0.038 | 0.075 | 0.188 | 0.750 | 1.88 | 7.50 | 15.00 | 30.00 |
| SM d18:1/18:1 | 0.0048 | 0.0096 | 0.019 | 0.038 | 0.096 | 0.384 | 0.96 | 3.84 | 7.68 | 15.35 |
| SHexCer d18:1_17:0 | 0.0031 | 0.0063 | 0.013 | 0.025 | 0.063 | 0.250 | 0.63 | 1.25 | 2.50 | 5.00 |
| MAG 18:1 | 0.0098 | 0.0196 | 0.039 | 0.078 | 0.196 | 0.785 | 1.96 | 7.85 | 15.70 | 31.40 |

**Supplemental Table S4.** Concentrations of deuterated internal standards used for Method Validation and sample analysis

| **Standard** | **Stock Conc. (µg/mL)** | **Final Conc (µM) in plasma** |
| --- | --- | --- |
| PC 15:0_18:1d7 | 100 | 1 |
| LPC 18:1d7 | 100 | 1.42 |
| PE 15:0_18:1d7 | 100 | 1.06 |
| LPE 18:1d7 | 100 | 1.55 |
| PG 15:0-18:1d7 | 100 | 0.99 |
| Lyso PG 17:0 -d5 ^a^ | 1000 | 1 |
| PI 15:0-18:1d7 | 100 | 0.89 |
| Lyso PI 17:0 d5 ^a^ | 500 | 2.5 |
| PS 15:0-18:1d7 | 100 | 0.97 |
| Lyso PS 17:0 -d5 ^a^ | 1000 | 2 |
| TAG 15:0-18:1d7-15:0 | 100 | 0.93 |
| DAG 15:0-18:1d7 | 100 | 1.28 |
| MAG 18:1d7 | 100 | 2.07 |
| CE 18:1d7 | 100 | 1.14 |
| SM d18:1d7/18:1 | 100 | 1.02 |
| PE P 18:1/18:1d9 | 1000 | 1 |
| PC P 18:1/18:1d9 | 1000 | 1 |
| LacCer d18:1d9/15:0 | 1000 | 1 |
| GalCer d18:1d7/13:0 | 1000 | 1 |
| GlcCer d18:1d7/15:0 | 1000 | 1 |
| Cer d18:1d7/15:0 | 100 | 1.42 |
| Cer d18:1d7/16:0 | 21.8 | 0.46 |
| Cer d18:1d7/18:0 | 11.5 | 0.23 |
| Cer d18:1d7/24:0 | 26.3 | 0.46 |
| Cer d18:1d7/24:1 | 13.1 | 0.23 |
| SHexCer d18:1d7/13:0 ^a^ | 1000 | 1 |
| FFA 18:0 d3 | 575 | 1 |
| Cholesterol-d7 ^b^ | 1000 | 2.5 |

^a^ Not included in the inter-assay validation.

^b^ Separate APCI analysis

**Supplemental Table S5.** Chromatographic separation conditions. For normal phase chromatography, a CORTECS HILIC column (90Å, 2.7 µm, 4.6 mm X 150mm, Waters) was used, applying the mobile phases A: n-hexane, B: 50/50 ACN/water containing 0.1% formic acid, C: acetone/dichloromethane (2:1, v/v), D: methanol. Hydrophilic interaction chromatography separation was performed on a HALO HILIC (90Å, 2.7 µm, 4.6 mm X 150 mm, Advanced Materials Technology), using the mobile phases A: water containing 15 mM ammonium acetate, B: 98.5/1.5 ACN/water containing 15 mM ammonium acetate.

**Supplemental Table S6.** Lipid extraction recoveries.

| **Lipid Class** | **Lipid** | **Absolute Recovery^a^** | **Standard deviation^b^** |
| --- | --- | --- | --- |
| TG | 14:0-13:0-14:0_TG-d5 (41:1) | 86 | 7 |
| TG | 14:0-15:1-14:0_TG-d5 (43:1) | 87 | 9 |
| TG | 14:0-17:1-14:0_TG-d5 (45:1) | 75 | 9 |
| TG | 16:0-15:1-16:0_TG-d5 (47:1) | 79 | 3 |
| TG | 16:0-17:1-16:0_TG-d5 (49:1) | 81 | 6 |
| TG | 16:0-19:2-16:0_TG-d5 (51:2) | 78 | 7 |
| TG | 18:1-17:1-18:1_TG-d5 (53:3) | 78 | 2 |
| TG | 18:1-19:2-18:1_TG-d5 (55:4) | 69 | 8 |
| TG | 18:1-21:2-18:1_TG-d5 (57:4) | 69 | 7 |
| TG | TAG15:0-18:1d7-15:0 | 97 | 10 |
| CE | 16:1d7 CE | 102 | 17 |
| CE | 18:1d7 CE | 100 | 8 |
| CE | 20:3d7 CE | 115 | 18 |
| CE | 22:4d7 CE | 91 | 6 |
| CE | CE 18:1d7 | 96 | 8 |
| DG | DAG 1,3-16:1d5 | 93 | 8 |
| DG | DAG 1,3-18:0d5 | 84 | 13 |
| DG | DAG 1,3-18:1d5 | 89 | 11 |
| DG | DAG 1,3-18:2d5 | 106 | 8 |
| DG | DAG 15:0_18:1d7 | 92 | 8 |
| MG | MAG 18:1d7 | 79 | 10 |
| FFA | FFA 18:0 d3 | 92 | 4 |
| SM | SM d18:1d9/18:1 | 91 | 7 |
| CER | Cer d18:1d7/15:0 | 97 | 4 |
| CER | Cer d18:1d7/16:0 | 97 | 3 |
| CER | Cer d18:1d7/18:0 | 94 | 8 |
| CER | Cer d18:1d7/24:0 | 96 | 8 |
| CER | Cer d18:1d7/24:1 | 91 | 3 |
| HexCer | GalCer d18:1d7/13:0 | 92 | 3 |
| HexCer | GluCer d18:1d7/15:0 | 91 | 5 |
| Hex2Cer | LacCer d18:1d7/15:0 | 92 | 7 |
| Sulfatide | Sulfo GalCer d18:1/13:0d7 | 77 | 3 |
| Sulfatide | N-C18:0-CD3-Sulfatide | 83 | 2 |
| PC | PC 15:0_18:1d7 | 92 | 2 |
| PC P | PC P-18:0_18:1d9 | 92 | 6 |
| PE | PE 18:1d7_15:0 | 92 | 4 |
| PE P | PE P-18:0_18:1d9 | 87 | 2 |
| PG | PG 18:1d7_15:0 | 84 | 4 |
| PI | PI 18:1d7_15:0 | 71 | 4 |
| PA | PA 15:0_18:1d7 | 76 | 4 |
| PS | PS 15:0_18:1d7 | 72 | 14 |
| LPI | LPI 17:0d5 | 13 | 2 |
| LPS | LPS 17:0d5 | 22 | 3 |
| LPC | LPC 18:1d7 | 92 | 1 |
| LPE | LPE 18:1d7 | 93 | 3 |
| LPG | LPG 17:0d5 | 45 | 1 |

PA ratio: LC/MRM (peak area of stable isotope labeled lipid)/(peak area of corresponding endogenous lipid)

^a^ Absolute recovery = (mean PA ratio of pre-spiked deuterated lipids)/(mean PA ratio of post-spiked deuterated lipids) *100

Endogenous lipids in human plasma for signal normalization: TAG 44:1_16:0, CE 16:1, DAG 16:0_18:1, MG 18:1, FFA 18:2, SM 36:2, Cer d18:1_16:0, HexCer d18:1_16:0, diHexCer d18:1_16:0, Sulfatide d18:1_16:0, PC 16:0_18:2, PE d18:1_16:0, PG 16:0_18:1, PI 16:0_18:1, PS 18:0_16:0, LPI 18:0, LPS 18:0, LPC 18:1. LPE 18:1 and LPG 18:1

^b^ Recovery SD = Standard deviation of PA ratio of pre-spiked deuterated lipids (n=4-5)/mean PA ratio of post-spiked deuterated lipids*100

**Supplemental Table S7.** Corrected signal drifting of SM d18:1/18:1 using multiple calibration curves and QCs (NIST 195 plasma).

| **QC** | **Date** | **All QCs back from curve 1 (µM)** | **All QCs back from curve 2 (µM)** | **QC1-5 from Curve1; QC6-10 from curve 2 (µM)** |
| --- | --- | --- | --- | --- |
| **Calibration curve 1** | **Y=0.73191x-0.01507, R_square = 0.99978, 1/x** | | | |
| QC1 | day1 10:34:26 PM | 5.382 | 3.894 | 5.382 |
| QC2 | day2 5:25:32 AM | 8.358 | 6.045 | 8.358 |
| QC3 | day2 2:56:34 PM | 7.272 | 5.259 | 7.272 |
| QC4 | day3 3:53:11 AM | 6.213 | 4.494 | 6.213 |
| QC5 | day3 10:21:22 AM | 6.879 | 4.976 | 6.879 |
| **Calibration curve 2** | **Y=1.010x-0.02023, R_square = 0.99801, 1/x** | | | |
| QC6 | day4 6:54:33 PM | 10.478 | 7.576 | 7.576 |
| QC7 | day 5 1:07:55 AM | 10.958 | 7.923 | 7.923 |
| QC8 | day 5 4:56:09 AM | 10.521 | 7.608 | 7.608 |
| QC9 | day 5 7:42:07 AM | 11.388 | 8.234 | 8.234 |
| QC10 | day 5 8:44:23 AM | 10.002 | 7.232 | 7.232 |
| **%RSD** |  | **25.0** | **25.0** | **12.6** |

**Supplemental Figure S1.** NPLC separation of *sn* positional isomers of DAG (A) and LPC (B). 1,2-DAG 15:0/18:0d7 in blue (MRM transition for 18:0d7), 1,2-DAG 16:0_18:1 in red (MRM transition for 16:0), 1,2**-**DAG 16:0_18:1 in green (MRM transition for 18:1) and 1,2-DAG 18:1_18:1 in grey (MRM transition for 18:1). About 10% of the 1,2-DAGs are detected as 1,3 isomers, which could be due to impurities or auto migration of the 1,2 standards to the more energetically favorable 1,3 form.

**Supplemental Figure S2.** ESI source temperature dependence. Phospholipids (A), sterol esters and glycerolipids (B), and sphingolipids (C) were monitored using NPLC at different ion source temperature. Peak areas of each lipid at 450°C and 600°C are normalized to the peak areas obtained at 300°C.

**Supplemental Figure S3**. In-source fragmentation of Sulfatide. In-source neutral loss of [M-H_2_O], [M-SO_3_] and [M-SO_3_-H_2_O] for [A]= SHexCer d18:1/13:0, [B]= SHexCer d18:1/17:0, [C]= SHexCer d18:1/24:1 (**A**). SHexCer d18:1d7/13:1 and GalCer d18:1d7/13:1 mixture analyzed by NPLC in positive ion MRM mode (**B**). SHexCer d18:1d7/13:1 (Rt 1.05 min) and GalCer d18:1d7/13:1 (Rt 4.0 min) mixture analyzed by HILIC in positive ion MRM mode (**C**).


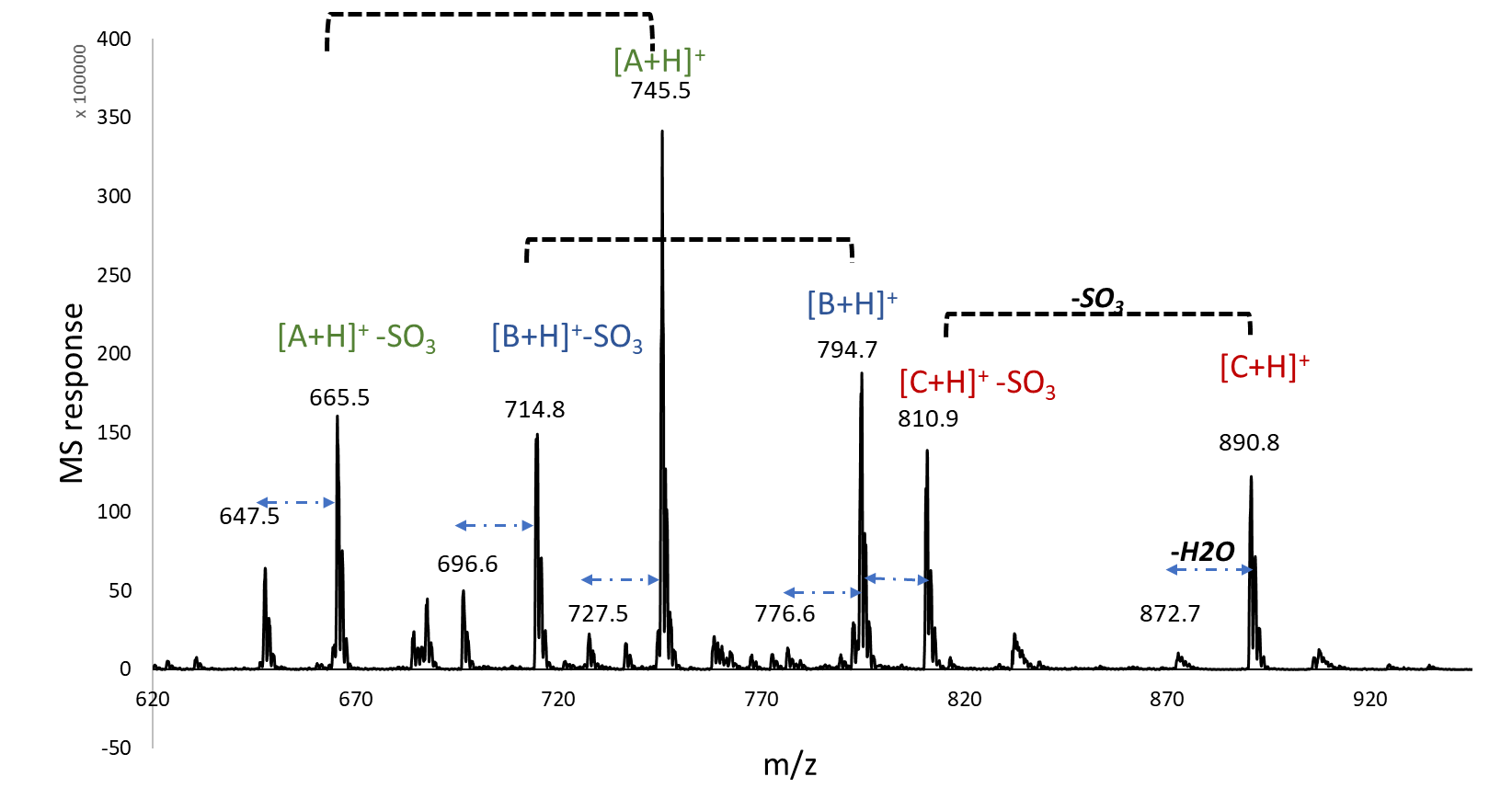
A

****B

**HILIC**

Positive MRM

665.5 → 271.2 (GalCer d18:1d7/13:1)

745.5 → 271.2 (SHexCer d18:1d7/13:1)

C

**NPLC**

Positive MRM

665.5 → 271.2 (GalCer d18:1d7/13:1)

745.5 → 271.2 (SHexCer d18:1d7/13:1)

**Supplemental Figure S4**. Effects of fatty acyl chain length and number of double bonds on the retention behavior. Retention times of LPC lipid species (relative to LPC 20:0) (A) and SM species (relative to SM d18:1_24:0) (B) extracted from mice plasma.

**A**.
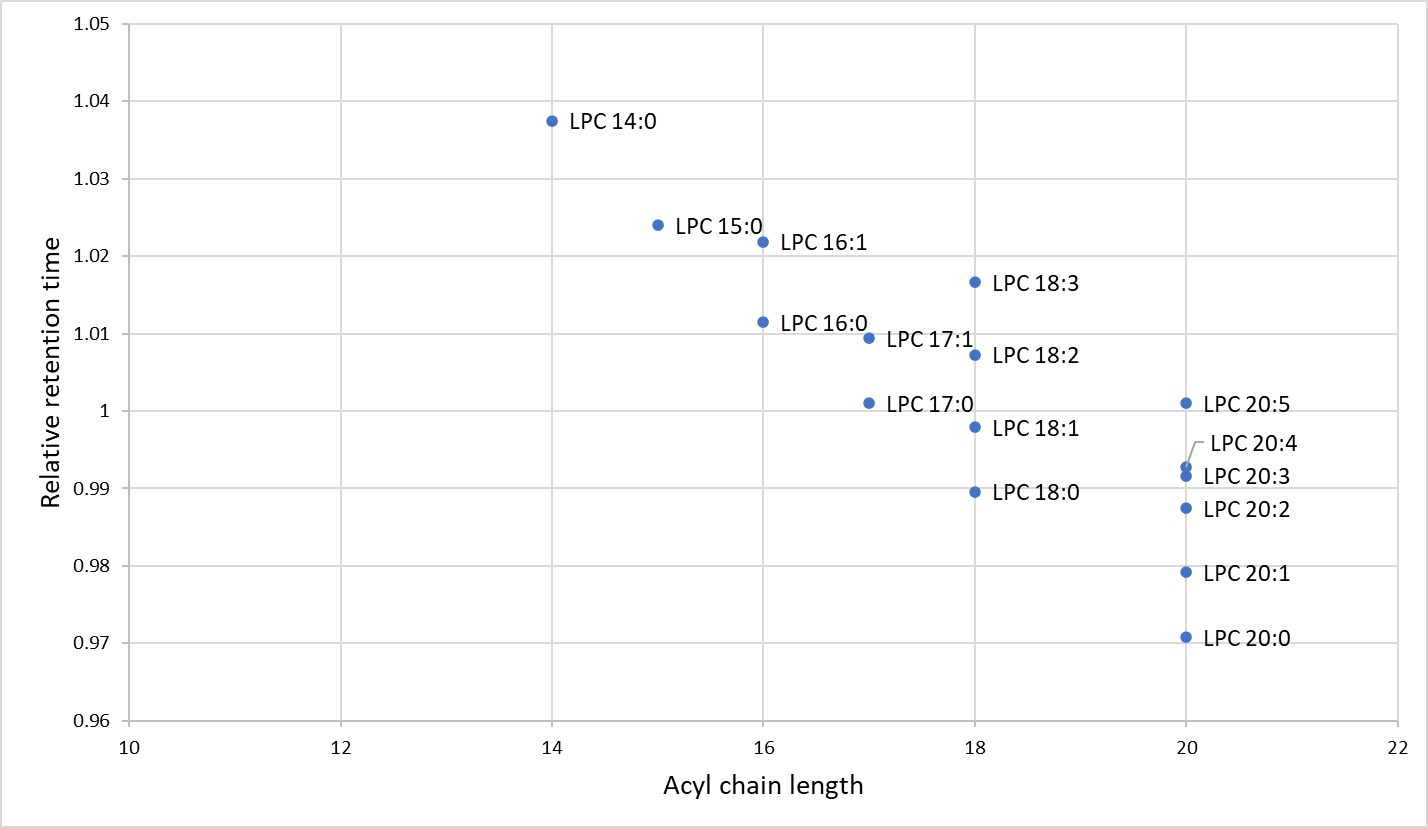


**B.**

**
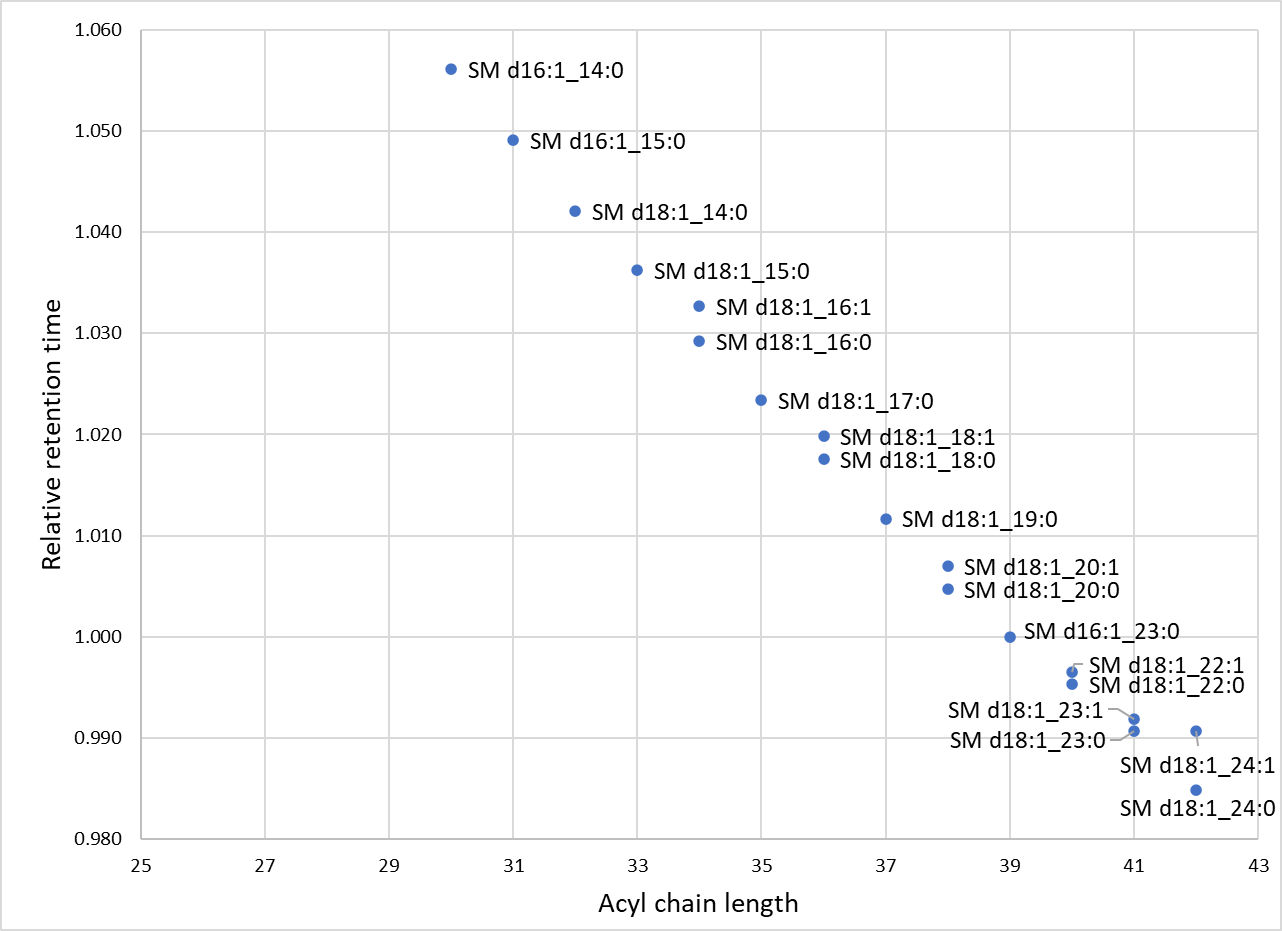
**

**Supplemental Figure S5.** *sn*-1 and *sn*-2 positional isomer quantification curves (n=3) from pure standards.

**A**: 1μM PE 16:0/18:1(n9) and 1μM PE 18:1(n9)/16:0 were mixed in different ratios by volume (V).

X-axis represents % PE18:1/16:0 (V(PE18:1/16:0)/[V(PE18:1/16:0)+V(PE16:0/18:1)]*100), and Y-axis %Peak (100* Peak area of MRM [PE-H]^-^→[FA18:1-H]^-^ / sum peak areas of {[PE-H]^-^ →[FA16:1-H]^-^ and [M-H]^-^→[FA18:1-H]^-^ }).

**B**: 1μM PC 18:1(n10)/16:0 and 1μM PC 16:0/18:1(n10) were mixed in different ratios by volume (V).

X-axis represents % PC18:1/16:0 (V(PC18:1/16:0)/[V(PC18:1/16:0)+V(PC16:0/18:1)]*100), and Y-axis %Peak area (100* Peak area of MRM [PC+CH_3_COO]^-^→[FA18:1-H]^-^ / sum peak areas of {[PC CH_3_COO]^-^ →[FA16:1-H]^-^ and [PC-CH_3_COO]^-^→[FA18:1-H]^-^}).

**A**

**B**

**Supplemental Figure S6.** Dilution linearity and extension of quantification dynamic range beyond ULOQ of standard curve using endogenous lipids. Linear fitting was performed to the peak area ratios (analyte to SIL IS) of 25x, 5x and undiluted NIST SRM1950 plasma (n=6 per dilution) and the average R^2^ from 46 lipid species and standard deviations were plotted (i.e. LPC 16:0, LPC 18:0, LPC 18:1, LPC 18:2, PC 14:0_16:0, PC 16:0_16:0, PC 16:0_22:6, PC 16:1_16:0, PC 18:1_14:0, PC 18:1_16:0, PC 18:1_18:0, PC 18:1_18:1, PC 18:2_14:0, PC 18:2_16:0, PC 18:2_16:1, PC 18:2_18:0, PC 18:2_18:1, PC 18:2_18:2, PC 20:3_16:0, PC 20:3_18:0, PC 20:3_18:1, PC 20:4_16:0, PC 20:4_18:0, PC 20:4_18:1, PC 22:5_16:0, PC 22:5_18:0, PC 22:6_18:0, PC O-32:0, PC O-36:0, PC O-38:2, PC O-38:7, PI 18:0_20:4, PI 20:4_18:0, SM d18:1/14:0, SM d18:1/16:0, SM d18:1/16:1 , SM d18:1/17:0, SM d18:1/18:0, SM d18:1/18:1, SM d18:1/20:0, SM d18:1/20:1, SM d18:1/22:0, SM d18:1/22:1, SM d18:1/24:0, and SM d18:1/24:1) (A). The upper limit of quantification from standard curve was 15.35 μM (**Table S-3**) for SM. As the peak area ratios of (endogenous SM d18:1/16:0 to SM 18:1d7/18:1) from 25x, 5x and undiluted NIST 195 plasma shows linear fitting, the ULQ can be extended to 25 μM (B).

**A.**

**B.**

**Supplemental Figure S7**. Correlation analysis against published studies. The quantities of molecular PC species in NIST SRM1950 were summed to represent sum PC species. **A.** Correlation analysis was performed against the sum PC quantities in NIST SRM1950 obtained by Bowden et al (Ref) (blue) and Ghorasaini et al (10.1021/acs.analchem.1c02826) (red). Linear regression was performed using Prism 9 to derive the R^2^ values. **B.** Quantities of the individual molecular species representing the sum PCs in NIST SRM1950.

**Supplemental Figure S8**. Alterations in lipid fatty acid distributions in composition correlated with GCS inhibition at 4 days and 4 weeks treatment. BZ1-dependent alterations in lipids comparing 4-day to 4-week treatments are expressed as both absolute concentrations as well as relative percent change from vehicle treated mice as baselines. Data are arranged to reflect the overall lipid homeostasis changes in phospholipids including sphingomyelin. Time-dependent decreases in SM, PC and PI species coincide with elevations of fatty acid selective and PE and PE-P species. Statistical significance was determined using unpaired t-test corrected for multiple comparisons by the Holm-Šídák method. * P < 0.05; ** P < 0.01; *** P < 0.001; **** P < 0.0001; ***** P < 0.00001; ****** P < 0.000001.

**REFERENCES**

1. Ståhlman, M., C. S. Ejsing, K. Tarasov, J. Perman, J. Borén, and K. Ekroos. 2009. High-throughput shotgun lipidomics by quadrupole time-of-flight mass spectrometry. *J. Chromatogr. B. Analyt. Technol. Biomed. Life Sci.* **877**: 2664–72.
